# Ran GTPase, an eukaryotic gene novelty, is involved in amphioxus mitosis

**DOI:** 10.1371/journal.pone.0196930

**Published:** 2018-10-09

**Authors:** Ugo Coppola, Filomena Caccavale, Marta Scelzo, Nicholas D. Holland, Filomena Ristoratore, Salvatore D’Aniello

**Affiliations:** 1 Biology and Evolution of Marine Organisms, Stazione Zoologica Anton Dohrn Napoli, Villa Comunale, Napoli, Italy; 2 Laboratory of Developmental Biology of Villefranche sur Mer, UMR7009 CNRS/UPMC Observatoire Océanologique, Villefranche sur Mer, France; 3 Marine Biology Research Division, Scripps Institution of Oceanography, University of California at San Diego, La Jolla, CA, United States of America; Academia Sinica, TAIWAN

## Abstract

Ran (ras-related nuclear protein) is a small GTPase belonging to the RAS superfamily that is specialized in nuclear trafficking. Through different accessory proteins, Ran plays key roles in several processes including nuclear import-export, mitotic progression and spindle assembly. Consequently, Ran dysfunction has been linked to several human pathologies. This work illustrates the high degree of amino acid conservation of Ran orthologues across evolution, reflected in its conserved role in nuclear trafficking. Moreover, we studied the evolutionary scenario of the pre-metazoan genetic linkage between *Ran* and *Stx*, and we hypothesized that chromosomal proximity of these two genes across metazoans could be related to a regulatory logic or a functional linkage. We studied, for the first time, *Ran* expression during amphioxus development and reported its presence in the neural vesicle, mouth, gill slits and gut corresponding to body regions involved in active cell division.

## Introduction

Ras-related nuclear protein (Ran) is an eukaryotic evolutionary conserved small GTPase representing the sole Ras superfamily member active in nucleus and traditionally considered a master regulator of nuclear trafficking [1,2]. Like other Ras proteins, it interacts with many cellular proteins comprising GTPase-activating protein (RanGAP), guanine-nucleotide-exchange factor (RanGEF) and GTP-Ran binding proteins, such as RanBP1 [3]. RanGEF catalyzes the GDP/GTP exchange while RanGAP promotes the GTP hydrolysis of Ran protein. In contrast, the cytoplasmic RanBP1 is an accessory protein that accelerates GTP hydrolysis carried out by RanGAP when other GTP-Ran binding proteins are absent [4]. A further relevant Ran accessory protein implicated in GTP hydrolysis is the importin β3, also named RanBP5, able to bind nuclear pore complexes [5]. Ran activity is crucial for nuclear import-export, and the presence of a GTP-Ran gradient across the nuclear envelope has been implicated in nucleo-cytoplasmic transport [6]. In addition, RanBP1 overexpression in murine fibroblasts and *Xenopus laevis* egg extracts has demonstrated the involvement of Ran pathway in mitotic progression [7,8]. The decrease of GTP-Ran levels in frog eggs resulted in disrupted spindle assembly, potentially connected to tubulin polymerization microtubule activities [8] and to DNA replication [9]. Moreover, it has been shown that Ran is involved in the stability of centrosomes [10].

In mouse embryos *Ran* is expressed in the nervous system with a strong accumulation in neural crest cells and sensory pits, as well as in the hematopoietic system with prevalence in blood islands [11]. The expression in mammalian nervous tissues is consistent with that observed in *Drosophila* and *Xenopus* [12,13]. In the prawn *Penaeus monodon Ran* transcripts are present in several body tissues and especially in developing ovary [14]. Recently, Ran has been described as a fundamental player in retinal cell proliferation and differentiation in zebrafish [15].

*Ran* has been associated to several human pathologies, like the rare X-linked neuron Kennedy’s disease caused by the expansion of the polyglutamine repeated region inside the androgen receptor, resulting in spinal and bulbar muscular atrophy [16]. Ran promotes bacteria infection through a modification of vacuole motility in Legionnaires’ disease, an atypical pneumonia caused by bacteria belonging to *Legionella* genus [17]. Moreover, *Ran* has been identified as a marker for the development of melanoma [18] and it is involved in teratocarcinoma, the most common testicular germ cell tumours [19], in serous epithelial ovarian tumour [20] and in lung cancer [21].

The present study reveals high conservation in the organization of the *Ran* locus during metazoan evolution as well as in the amino acid sequences of Ran proteins in a spectrum of eukaryotes. Special attention is given to the structure and function of Ran in the European amphioxus, *Branchiostoma lanceolatum*, in light of its key phylogenetic position within the phylum *Chordata*. Importantly, amphioxus Ran is conspicuously expressed in the developing neural and endodermal regions undergoing intensive cell division. This pattern is consistent with a key role for amphioxus Ran in mitotic dynamics in agreement with previous studies of the involvement of vertebrate Ran in cell proliferation during normal development and neoplastic growth.

## Results

### Ran protein sequence conservation

In order to study the evolution of nuclear-specific Ran, we carried out a detailed search in representatives of several available genomes and we did not find any Ran protein in Eubacteria and Archaea sequenced until now. This could suggest that *Ran* genes are an exclusive feature of Eukaryotes. We retrieved Ran proteins from available genomes encompassing: unicellular eukaryotes (*Monosiga brevicollis*, *Capsaspora owczarzaki*, *Fusarium migrarium*, *Saccharomyces cerevisiae*, *Puccinia striiformis*, *Fragilariopsis cylindrus*), plants (*Arabidopsis thaliana*, *Oryza sativa*), sponges (*Amphimedon queenslandica*), placozoans (*Trichoplax adhaerens*), ctenophores (*Mnemiopsis leidyi*), cnidarians (*Exaiptasia pallida*, *Nematostella vectensis*), arthropods (*Drosophila melanogaster*, *Limulus poliphemus*, *Daphnia magna*), nematodes (*Caenorhabditis elegans*), mollusks (*Lottia gigantea*, *Crassostrea gigas*, *Octopus bimaculoides*), annelids (*Capitella teleta*), brachiopods (*Lingula anatina*), priapulids (*Priapulus caudatus*), echinoderms (*Strongylocentrotus purpuratus*), hemichordates (*Saccoglossus kowalevskii*), cephalochordates (*Branchiostoma floridae*, *Branchiostoma lanceolatum*), urochordates (*Oikopleura dioica*, *Ciona robusta*, formerly known as *Ciona intestinalis* type A [22]), and vertebrates (*Lethenteron japonicum*, *Callorhinchus milii*, *Latimeria chalumnae*, *Lepisosteus oculatus*, *Scleropages formosus*, *Danio rerio*, *Salmo salar*, *Xenopus tropicalis*, *Gallus gallus*, *Anolis carolinensis*, *Homo sapiens*) (S1 File).

**Fig 1:**
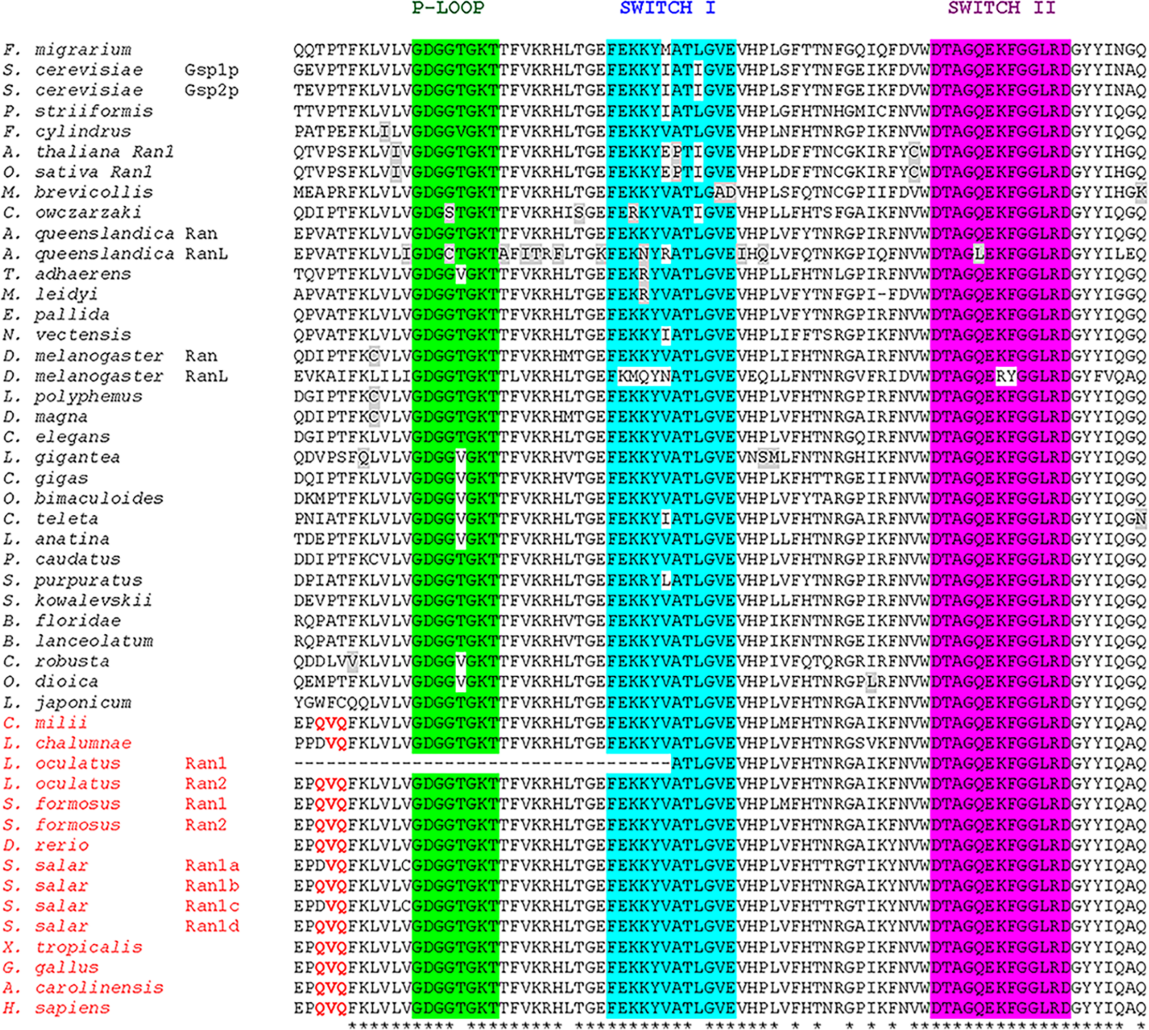
Ran protein alignment. This picture shows the alignment of Ran proteins from unicellular eukaryotes to human, focused on the three main Rab domains: P-Loop (green), Switch I (blue) and Switch II (magenta). Asterisks have been used to point out the total amino acid conservation (81%, with ≤ 3 residues change). We highlighted in red the gnathostome-specific amino acid changes.

Most of the surveyed species possess a single-copy gene, except the yeast *S*. *cerevisiae*, the demosponge *A*. *queenslandica*, the fly *D*. *melanogaster*, the spotted gar *L*. *oculatus* and the Asian Arowana *S*. *formosus*, in which we found a second protein (Fig 1). Moreover, we found four Ran proteins in salmon *S*. *salar*. Given that some vertebrates possess more than one Ran, we performed a phylogenetic tree suggesting the presence of two distinct *Ran* paralogues in gnathostomes (S1 Fig), that we called *Ran1* and *Ran2*. The unique Ran of tetrapods, coelacanth and ghost shark belongs to *Ran2* clade, whereas eutelosts (zebrafish, salmon) possess only *Ran1*. On the other hand, spotted gar and *Asian arowana* retain both *Ran* genes (S1 Fig).

The alignment of 48 proteins from 40 species (Fig 1) revealed a high degree of Ran primary structure conservation across eukaryotes. Our analysis has been focused on a region of 79 amino acids comprising well-known functional Rab domains (P-Loop, Switch I, Switch II) with a very high sequence homology from unicellular eukaryotes to vertebrates (81%, with ≤ 3 residues change). Furthermore, we found that most of gnathostomes Ran proteins exhibit three conserved amino acid residues (QVQ) upstream to the P-Loop domain, which could confer an additional cellular function. The only exception was observed in coelacanth Ran and in salmon Ran1a and Ran1c, which have DVQ in place of QVQ.

Our findings indicate a strong degree of Ran conservation during evolution and a scenario of genome duplications and independent losses in vertebrates.

### *Ran*-*Syntaxin* microsyntenic pair evolution

**Fig 2:**
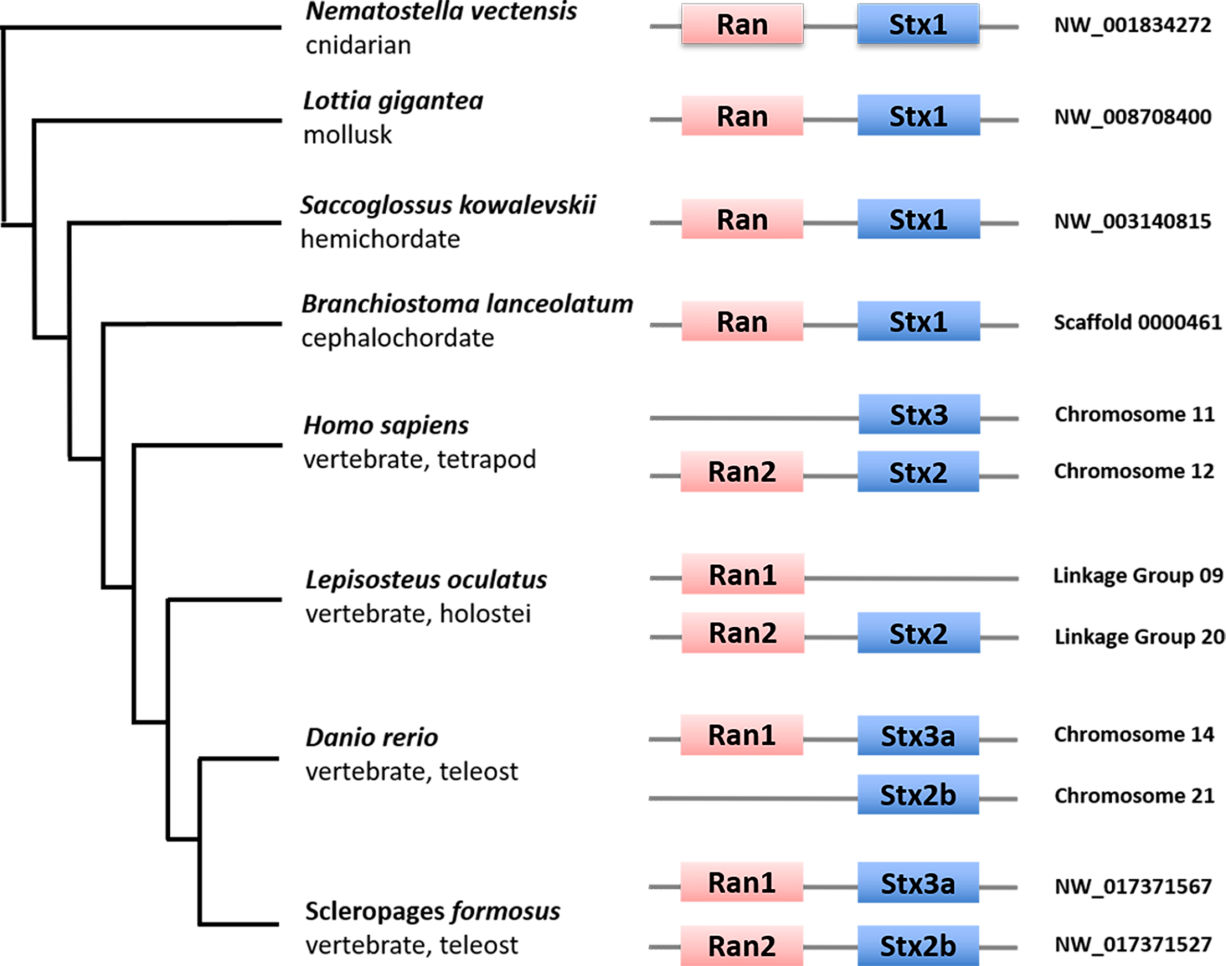
Conserved microsynteny between *Ran* and *Stx* genes during evolution. The scheme demonstrates the ultra-conserved genomic neighborhood between distinct *Ran* (pink boxes) and *Stx* (blue box) genes among distantly related species, showing how the ancestral genomic locus results conserved from protostomes to vertebrates. The Ran genomic *loci* of *Callhorhincus milii* (NW_006890249.1, *Ran2*) and *Latimeria chalumnae* (Scaffold JH126570.1, *Ran2*) show the same organization of human (data not shown).

In order to gain insights on Ran evolutionary history, we investigated its genomic locus in selected eukaryotic genomes. Irimia *et al*. [23] have identified an ultra-conserved gene duplet formed by *Ran* and *Syntaxin* (*Stx*), an important family of Q-SNARE proteins implicated in exocytosis [24]. Here, we updated the evolutionary scenario of this genomic locus by surveying more vertebrate species (gar *L*. *oculatus*, arowana *S*. *formosus*, and salmon *S*. *salar*). The microsyntenic pair in analysed invertebrates (sea anemone *N*. *vectensis*, limpet *L*. *gigantea*, acorn worm *S*. *kowalevskii* and amphioxus *B*. *lanceolatum*) is formed by *Ran* and *Stx1* (Fig 2). On the other hand, gnathostomes exhibit a different genomic combination of *Ran* and *Stx* genes. In order to clarify the origin of *Stx* and *Ran* duplet, we performed a phylogenetic analysis of Stx proteins (S2 Fig) and a syntenic survey in jawed vertebrates (S3 Fig, S4 Fig). The Maximum-Likelihood phylogenetic reconstruction showed the expansion of Stx family in vertebrates and the common evolutionary origin of *Stx* members flanking *Ran* genes (*Stx1*, *Stx2* and *Stx3*) (Fig 2). Stx proteins included in the tree have been listed in the S2 File.

As previously shown [23], *Ran* in invertebrates is always found associated to *Stx1*, while human gene is linked with *Stx2* (Fig 2). Additionally, we have found that the unique *Ran* of zebrafish *Danio rerio* is coupled with *Stx3a* (Fig 2). In the genomes of *L*. *oculatus* [25] and *S*. *formosus* [26] we found that both *Ran* genes are coupled on distinct scaffolds with *Stx2* and *Stx3a*, respectively (Fig 2). The analysis of *Ran* genomic loci in different vertebrates (human, spotted gar, Asian arowana, zebrafish, salmon) revealed a high degree of synteny (Fig 2, S3 Fig, S4 Fig) clarifying the orthology among multiple *Ran* and *Stx* genes (Fig 2): in particular we discovered that *Ran1* is always associated with *Stx3*, while *Ran2* with *Stx2* (S3 Fig) and they probably derive from a duplicative event at the stem of vertebrates [27,28]. Moreover, the syntenic survey found four *Ran1* loci in *S*. *salar* genome (S4 Fig), as an effect of teleost and salmonid extra genome duplications [29,30] and the loss of *Ran2* in euteleosts (S3 Fig and S4 Fig). Altogether, our data suggest the existence of the ancient chromosomal linkage between *Ran* and *Stx* genes, which in vertebrates has been affected by distinct genome duplications.

### *Ran* expression pattern in amphioxus

**Fig 3:**
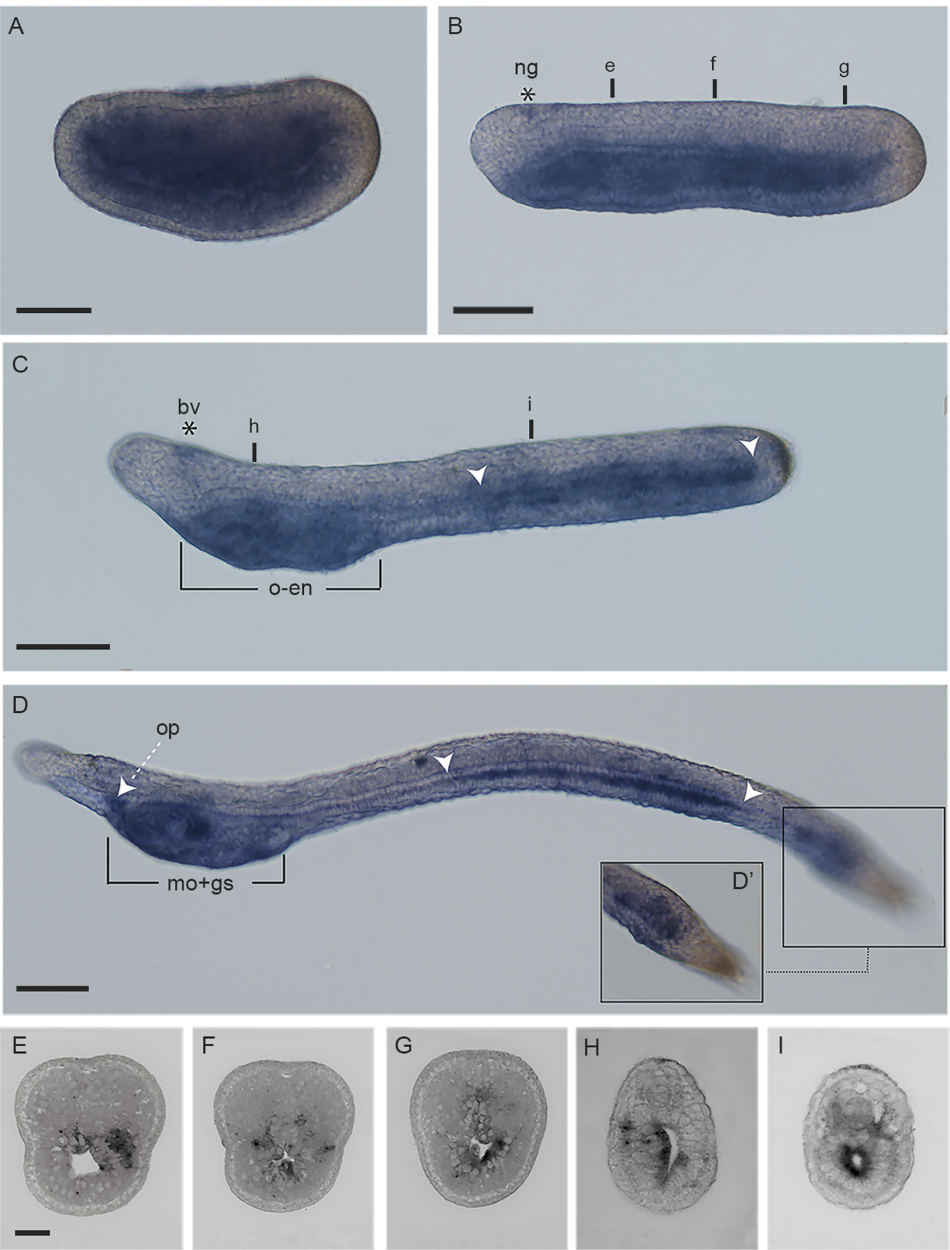
*Ran* expression pattern in amphioxus *Branchiostoma lanceolatum* embryos and larvae. A) middle neurula; B) late neurula; C) pre-mouth larva; D) 3 dpf larva; D’) enlargement of the 3 dpf larva’s tail. E) section of the late neurula in B at the level “e”. F) section of the late neurula in B at the level “f”. G) section of the late neurula in B at the level “g”. H) section of the pre-mouth in C at the level “h”. I) section of the pre-mouth in C at the level “i”. Abbreviation: ng, neural grove; bv, brain vesicle; o-en, oral endoderm; op, pre-oral pit; mo-gs, mouth and gill slits. White arrowheads indicate the anterior and posterior limits of Ran expression in gut. In all images the anterior is to the left and dorsal to the top. Scale bars: 60 μm in A-B-C-D; 15 μm in E.

The *Ran* expression pattern during *B*. *lanceolatum* development was analysed by whole mount *in situ* hybridization (Fig 3). At middle neurula stage (24 hpf), *Ran* mRNA has been detected prevalently in endoderm and mesoderm (Fig 3A). At late neurula (30 hpf), labeled cells are still present in endoderm in the ventral part of the embryos (Fig 3B, sections in Fig 3E, 3F and 3G), and Ran expressing cells have also been found close to neural groove (Fig 3B). Pre-mouth larvae (48 hpf) present a very faint *Ran* expression in brain vesicle (Fig 3C), in oral endoderm corresponding to the pharyngeal area, (Fig 3C and section in 3H) in gill slits and in the mid- and hindgut (white arrowheads, Fig 3C and section in 3I). At larval stage (72 hpf), *Ran* is expressed in pre-oral pit (white arrow), mouth and first gill slit, in midgut plus the rostral part of hindgut (white arrowheads) (Fig 3D). The region of the tailbud is also positively labeled as shown in Fig 3D’.

**Fig 4:**
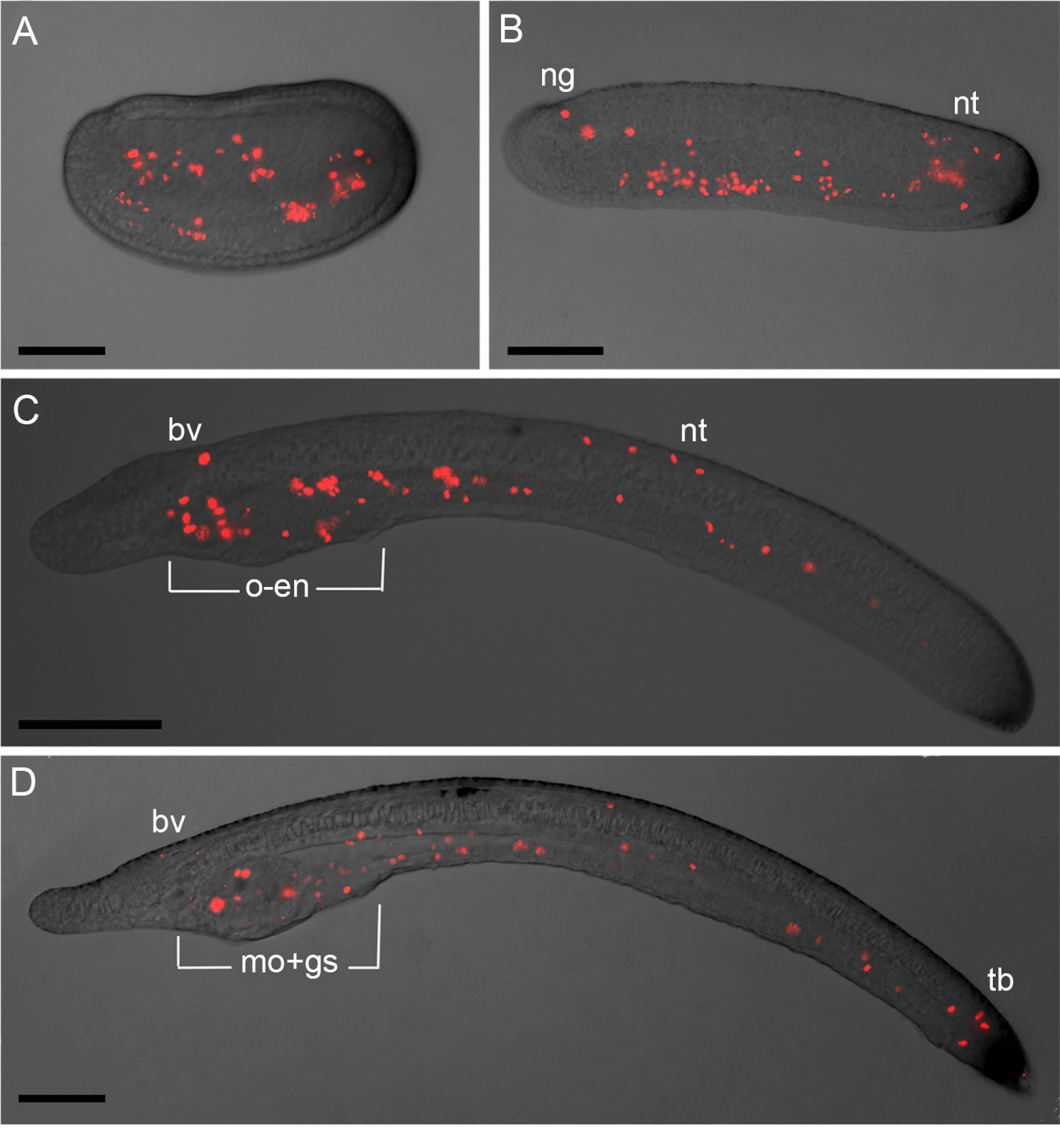
PHH3 antibody immunostaining in amphioxus *Branchiostoma lanceolatum* embryos and larvae. A) middle neurula; B) late neurula; C) pre-mouth larva; D) 3 dpf larva. Abbreviation: ng, neural grove; nt, neural tube; bv, brain vesicle; o-en, oral endoderm; mo-gs, mouth and gill slits; tb, tailbud. In all images the anterior is to the left and dorsal to the top. Scale bars: 60 μm.

Since a key role of Ran in cell divisions has been proposed, we performed immunostaining using PHH3 [Phospho-Histone H3 (Ser10)] antibody as a mitosis-specific marker in amphioxus to understand if there is a positive correlation of its expression pattern with cellular mitotic activity during development (Fig 4). PHH3 is expressed in proliferating cells and is important for the proper initial chromosomal condensation and segregation [31]. At middle neurula, the endoderm and somitic mesoderm cells are PHH3-positive, while ectodermal cells showed no evidence of proliferation (Fig 4A). Embryos at late neurula stage showed several positive cells in the endoderm, in the neural groove and in the posterior neural tube (Fig 4B). Later in development, at pre-mouth larvae, mitotic cells are visible in the oral endoderm region and in the neural tube (Fig 4C). At mouth-larva, PHH3 positive cells are present in the mouth, in the gill slits region and also along the gut (Fig 4D). Furthermore, few mitotic cells are visible in the most caudal part of the neural tube, and in the tailbud region, and residual signal is also detectable in the brain vesicle (Fig 4D). We observed that PHH3 expression profile varies slightly at different amphioxus specimens at the same developmental stage, as can be seen in additional pictures in S5 Fig. PHH3-positive cells pattern seemed to be faithfully superimposable to the dividing cells profile obtained in a previous study by using Bromodeoxyuridine [32], a synthetic analogue of thymidine, commonly used for detection of proliferating cells *in vivo*. In summary, we here described the *Ran* expression profile in embryos and larvae by whole mount *in situ* hybridizations, and compared it with PHH3 in order to inquire its involvement in cellular division in amphioxus.

## Discussion

Ran is an atypical small GTPase that is localized exclusively in nucleus and usually linked to macromolecular transport through nuclear pores. This protein plays a pivotal role in diverse mitotic steps in mammalian cells, like spindle formation and chromosome separation [33], and has been implicated in diverse human pathologies (detailed above in introduction).

Since Ran is so important for aspects of eukaryotic cell behaviour, we inquired into its molecular history broadly in Eukarya domain (Fig 1). Except few unrelated duplications, we found a single Ran from unicellular eukaryotes to higher plants and vertebrates, with a high degree of amino acid identity across tree of life. All the analysed taxa, in fact, possessed a Ran protein with an outstanding sequence conservation suggesting the importance of the evolutionary preservation of Ran role in cellular physiology. On the other hand, the fact that this protein has never been described in bacteria, despite extensive searches, speaks in favour of Ran advent in unicellular eukaryotes, consistently with a series of roles strictly related with nuclear functioning. The protein alignment hinted at some retained amino acid residue changes (QVQ) outside the fundamental domains in gnathostome Ran proteins, which indicates that there may be an unknown functional protein adaptation in this clade. Interestingly, the Ran of coelacanth and two Ran proteins of *S*. *salar* (Ran1a, Ran1c) show partial conservation of this amino acid stretch (DVQ), suggesting a possible diversification during evolution.

Additionally, if one considers that gene order on chromosomes is non-random [34], gene clusters can provide important insights into the evolution of genomes and their regulatory mechanisms. The physical linkage between *Ran* and *Stx*, two unrelated genes, belongs to a list of duplets unchanged across over 600 million years during evolution [23]: they form a conserved ancestral microsyntenic pair already present in the cnidarian-bilaterian ancestor. Our phylogeny evidences the common evolutionary origin for Syntaxins genes, i.e. *Stx1*, *Stx2* and *Stx3*, which flank *Ran* genes in different animal lineages; moreover *Stx1* is the unique gene present in non-vertebrate metazoans (S2 Fig).

Our phylogenetic and syntenic approach demonstrated that *Ran1* and *Ran2* of the gar and the arowana are paralogues deriving from whole-genome duplication at the root of vertebrate radiation [27,28]. The analysis of genomic loci of *Ran* genes showed that chondrichthyes and sarcopterygians have lost the *Ran1*, while zebrafish has lost *Ran2* (Fig 2, S1 Fig, S2 Fig). In light of the *Osteoglossomorpha* and *Holostea* data we inferred the actinopterygian ancestor possessed both the *Ran* genes, and *Ran2* was subsequently lost before the emergence of euteleosts (S1 Fig, S3 Fig). We found four *Ran* genes in *S*. *salar*, which previously was thought to have only two *Ran* [35] and this is probably due to teleost-specific (3R) and salmonid-specific fourth vertebrate whole-genome duplication (Ss4R) (S1 Fig) [29,36] [30]. The phylogeny and synteny demonstrated that salmon possess only *Ran1* paralogues (S1 Fig, S4 Fig): this fits well with the concept of *Ran1* as the unique gene retained in the ancestor of euteleosts. Since in extant analysed species *Ran1* is always associated with *Stx3* and *Ran2* with *Stx2* we hypothesized the existence of an ancestral linkage between a *Ran1/2* and a *Stx2/3* (S3 Fig, S4 Fig). Nevertheless, this gene duplet in gnathostomes has been strongly influenced by genome duplications and distinct gene losses whose impact should be further investigated [37].

The conserved *Ran-Stx* microsynteny might be due to regulatory logics: for instance, this linkage could be based on *cis*-Acting Transcriptional Repression [38]. Conversely, it has been proposed that Stx3 binds to RanBP5 in the nucleus interacting with several transcription factors [39], suggesting that Ran-Stx genomic proximity subtend a functional connection between Ran and Stx.

The amphioxus expression profile during embryogenesis could help our understanding of *Ran* evolutionary landscape in chordates, since cephalochordates are the closest living relatives of the ancestral chordate [40]. Our *in situ* hybridizations, in fact, showed *Ran* expression in several amphioxus tissues as mouth, gill slits, gut, and nervous system, whereas it is absent in epidermal cells: probably this is correlated with its multiple roles in the cell physiology. In particular, Ran is known to orchestrate multiple stages of the cell cycle including spindle assembly and nuclear envelope rearrangements, congruent with its important role in cell proliferation. Immunostaining with the proliferation marker PHH3 showed that cells of different embryonic structures are in active proliferation. It is also interesting to note that *Ran* mRNA expression is confined to structures known for their high proliferating rate: this suggests its possible function in cell division during amphioxus development. Interestingly, *Ran* absence in amphioxus epidermis could be associated to the strong expression of *Dral* proliferation suppressor in epidermal cells of late embryonic and early larval stages [41]. Moreover, *Ran* expression pattern in amphioxus is closely comparable to expression profiles previously observed in other animal models, supporting the concept of its determinant role in mitosis during evolution.

## Conclusions

Phylogenetic and syntenic survey shed light on the conservation of Ran from unicellular eukaryotes to vertebrates, with the preservation of protein sequence possibly linked to its role in mitosis. Moreover, we highlighted the presence of *Ran* in amphioxus proliferating tissues during embryogenesis. Inter alia, we expanded the current knowledge about the retained pre-metazoan genetic linkage between *Ran* and *Stx* genes, likely under strong stabilizing selection in distantly related lineages. Our findings unravelled a complex evolutionary scenario for *Ran-Stx* duplet, which has been heavily impacted by genome duplications and gene losses in vertebrates that needs further investigations in future.

## Materials and methods

### Molecular evolution analysis

The sequences employed for the evolutionary survey were retrieved from the NCBI, Ensembl and JGI databases. Human RAN was the initial query sequence employed for tBlastn searches [42] from unicellular eukaryotes to vertebrates, and reciprocal Blasts were carried out. The identity of Ran proteins was further assessed by using the domain bank of PROSITE database [43] and manually aligned as in Coppola *et al*. [44]. Synteny between *Ran* and *Stx* was mapped manually through the examination of public databases (Ensembl, NCBI).

The Stx and Ran proteins were aligned employing Clustal Omega [45] and the phylogenetic reconstructions were computed with the Maximum Likelihood (ML) estimation using MEGA6 with 1,000 replicates and the WAG matrix (γ = 4 and proportion of invariable sites = 0.4) [46] and the graphical representation was performed with Dendroscope [47]. *B*. *lanceolatum* sequences and genomic loci were annotated using the genome draft, kindly provided by the *Branchiostoma lanceolatum* genome consortium.

### Ethics statement

*Branchiostoma lanceolatum* (amphioxus) were collected in the Gulf of Napoli, Italy (latitude 40°48’33”N and longitude 14°12’55”E) from a location that is not privately-owned or protected, according to the authorization of Marina Mercantile (DPR 1639/68, 09/19/1980 confirmed by D. Lgs. 9/01/2012 n.4). The field studies did not involve endangered or protected species. All animal procedures were in compliance with the guidelines of the European Union.

### Amphioxus embryo collection

Adult amphioxus (*Branchiostoma lanceolatum*) were maintained in an open seawater circulation aquaculture system under a 14 hours light/10 hours dark cycle. Animals were reared in tanks at 16–20°C with 10 cm of sand from the collection site and fed daily using a mix of two unicellular algae: *Rhodomonas baltica* and *Isochrysis galbana*. Spawning events were induced indoor in late spring by employing a thermic shock as described by Fuentes *et al*. [48]. After *in vitro* fertilization, embryos were cultured in 0.22 μm filtered seawater at 18°C and at desired developmental stages were fixed with 4% paraformaldehyde (PFA) in MOPS buffer overnight at 4°C, and then stored in 70% ethanol at -20°C.

### Whole-mount *in situ* hybridization

Whole-mount *in situ* hybridization experiments were performed essentially as described in Annona *et al*. [49]. Briefly, embryos were rehydrated in phosphate buffered saline solution containing 0.1% Tween-20 (PBT) and treated with Proteinase K (5 μg/ml) to facilitate riboprobe penetration; the reaction was stopped by adding 4 μl of 10% glycine and then washed with 2 mg/ml glycine in PBT. The embryos were re-fixed in PBT containing 4% of PFA for 1 h at room temperature, subsequently washed in 0.1M triethanolamine and then in 0.1 M triethanolamine plus acetic anhydride, to prevent the formation of non-specific background. Embryos were washed with PBT several times and hybridized with the probe by shaking at 65°C overnight in DEPC-H_2_O hybridization buffer (50% deionized formamide; 100 μg/ml Heparin; 5X SSC; 0.1% Tween-20; 5 mM EDTA; Denhardt’s 1 mg/ml; yeast RNA 1 mg/ml). The day after, several washes were performed with decreasing concentration of SSC in 50% formamide/dH_2_O at hybridization temperature (from 5X to 2X), then a RNAse digestion was conducted at 37°C. Then, additional washes were carried out at room temperature using decreasing concentrations of SSC in dH_2_O (from 2X to 0.2X). Embryos were incubated overnight in primary antibody (anti-DIG AP, Roche), pre-adsorbed at 1:3000, with rocking at 4°C. The signal was revealed at room temperature using BM-Purple substrate (Roche). When the signal was deemed appropriate, several washes in PBT were performed before the post-fixation of embryos in PFA 4% for 20 minutes. Embryos were mounted in 80% glycerol in PBS, and photographed as whole mounts under the imaging system LEICA DMI6000B. Subsequently the embryos were counterstained in Ponceau S, embedded in Spurr’s resin, and prepared as sections for light microscopy [50].

### Whole-mount immunostaining

Embryos, fixed as in the preceding paragraph, were rehydrated in PBT and blocked for 1 h at room temperature in 2 mg/mL BSA, 10% goat serum in PBT 1X (Blocking solution). The embryos were incubated overnight at 4°C in rat Phospho anti-Histone H3 (PHH3) (Ser10) antibody (Upstate Biotechnology), diluted 1:250 in blocking solution. After labeling with the primary antibody, the specimens were rinsed in seven 20-min changes of PBT. Then a 1:500 dilution of the secondary antibody [Alexa Fluor 555 goat anti-rat IgG heavy and light chain (Thermo Fisher Scientific)] for 2 h at 4°C. The labeled specimens were rinsed in seven 20-min changes of PBT, mounted in 80% glycerol in PBS, and imaged by Axio Imager with ApoTome (Zeiss).

## Supporting information

S1 Fig Phylogenetic reconstruction of Ran proteins. The Maximum Likelihood (ML) tree indicates the existence in gnathostomes of Ran1 (orange box) and Ran2 (red box). The Ran of *Latimeria chalumnae* (Ran2) has been excluded from the tree for its divergence. Values at the branches indicate replicates obtained using the ML estimation method.
(PDF)

S2 Fig Phylogenetic reconstruction of Syntaxin family. In particular, the Maximum Likelihood (ML) tree evidences a common evolutionary origin for Stx1 (blue box), Stx2 (light blue box) and Stx3 (violet box). Values at the branches indicate replicates obtained using the Maximum Likelihood estimation method.
(PDF)

S3 Fig Analysis of *Ran* genomic *loci* in four vertebrates: *Homo sapiens* (chromosome 11 and chromosome 12), *Lepisosteus oculatus* (LG9 and LG20), *Scleropages formosus* (NW_017371567 and NW_017371527), *Danio rerio* (chromosome 14 and chromosome 8). A color code has been used to represent orthologue genes.
(PDF)

S4 Fig Ran genomic *loci* in *Salmo salar* (ssa04, ssa05, ssa08, ssa09, ssa24). (PDF)

S5 Fig Collection of two series of embryos (A’-A”, B’-B”, C’-C”, D’-D” represent distinct specimens) showing slightly different PHH3 immunolocalization signals (red). Scale bars: 60 μm. Embryos orientation: anterior to the left, dorsal to the top.
(PDF)

S1 File Database of sequences employed in Ran protein alignment of Fig 1 and Ran phylogeny of S1 Fig. (PDF)

S2 File Database of sequences used in Stx phylogeny of S2 Fig. (PDF)
