# Supplementary figures and images for: Ran GTPase, an eukaryotic gene novelty, is involved in amphioxus mitosis
Source: PLoS One. 2018 Oct 9;13(10):e0196930. doi: 10.1371/journal.pone.0196930 (PMC6177115; doi:10.1371/journal.pone.0196930)

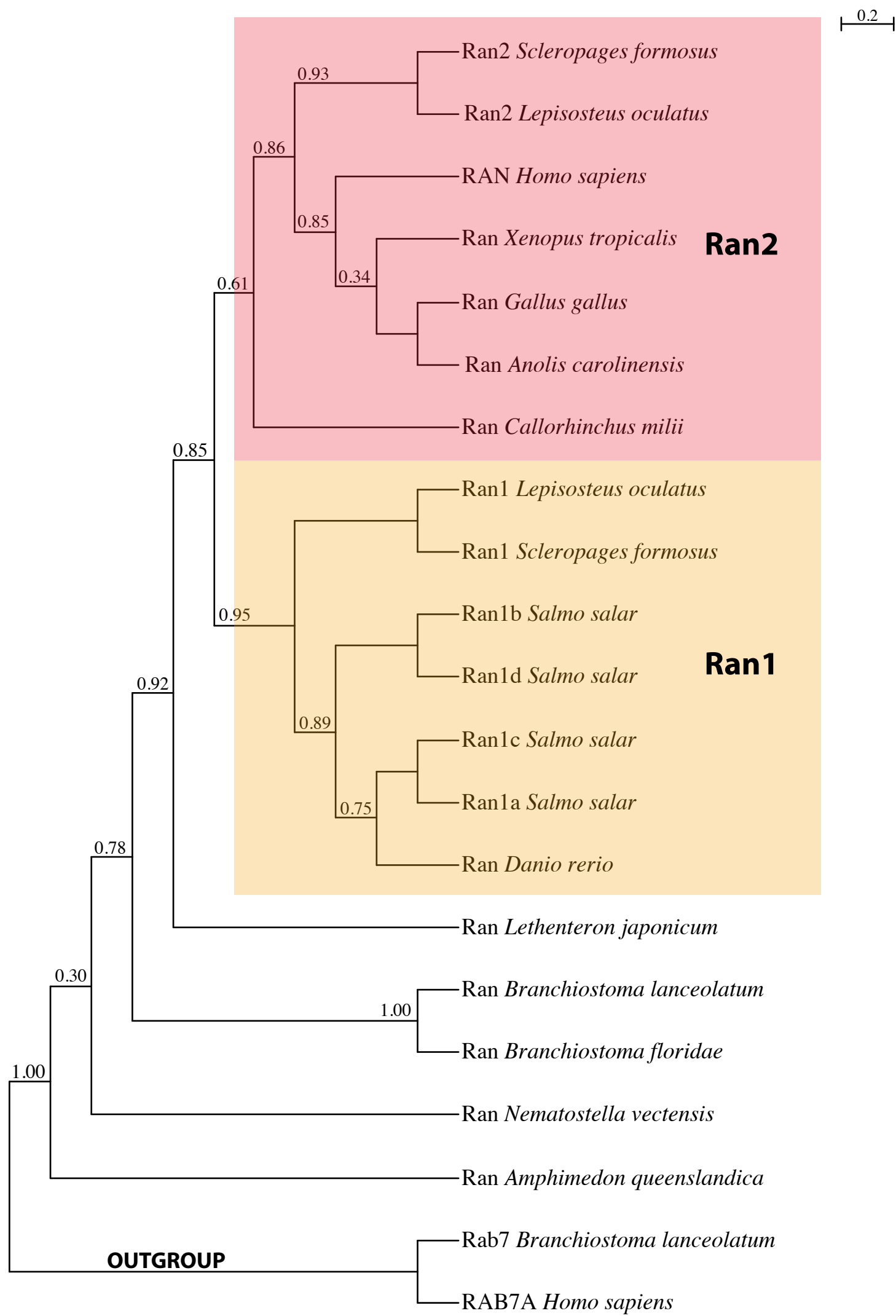

Supplement: S1 Fig — The Maximum Likelihood (ML) tree indicates the existence in gnathostomes of Ran1 (orange box) and Ran2 (red box). The Ran of Latimeria chalumnae (Ran2) has been excluded from the tree for its divergence. Values at the branches indicate replicates obtained using the ML estimation method. (PDF) [file pone.0196930.s001.pdf]

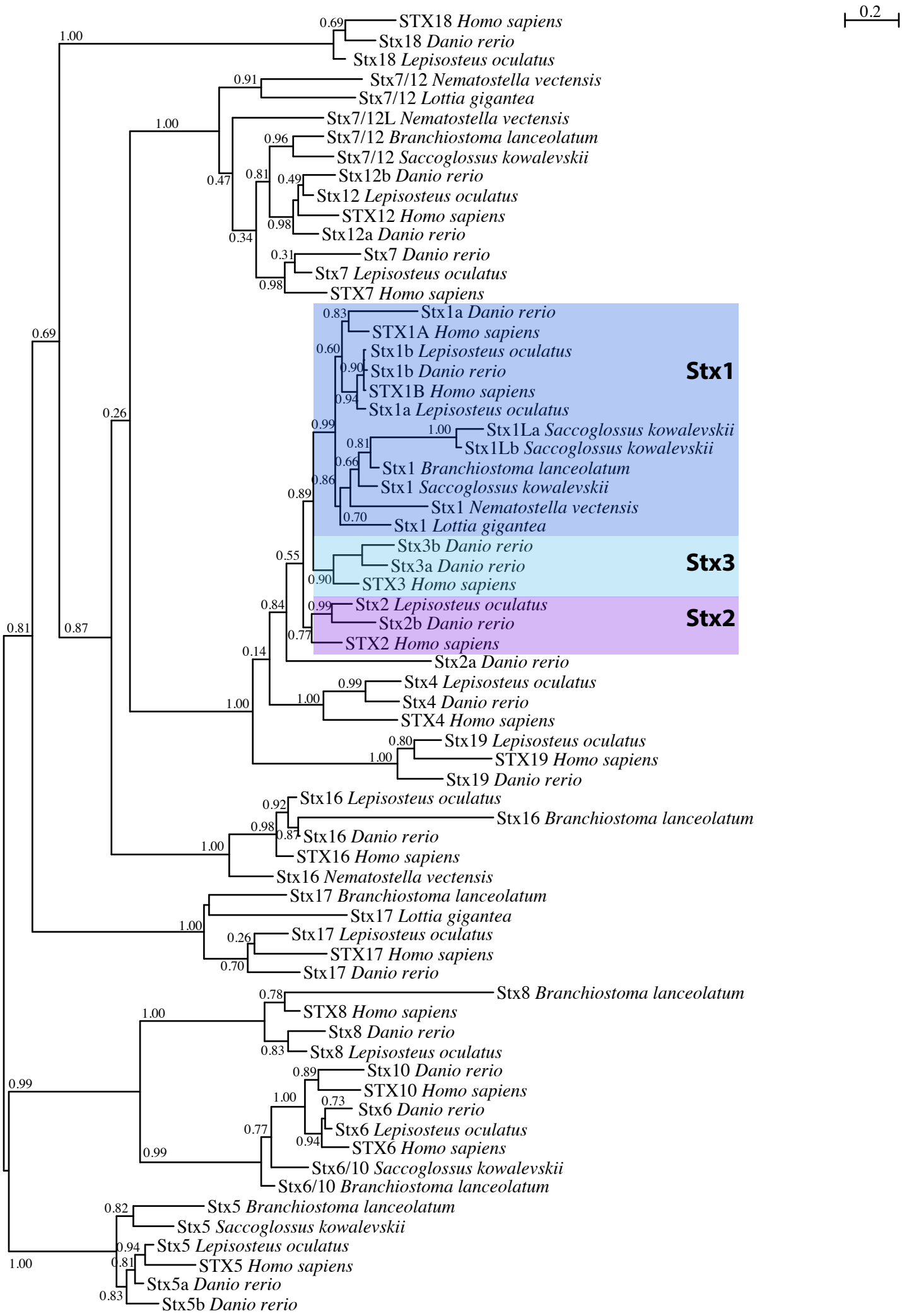

Supplement: S2 Fig — In particular, the Maximum Likelihood (ML) tree evidences a common evolutionary origin for Stx1 (blue box), Stx2 (light blue box) and Stx3 (violet box). Values at the branches indicate replicates obtained using the Maximum Likelihood estimation method. (PDF) [file pone.0196930.s002.pdf]

S3 Fig

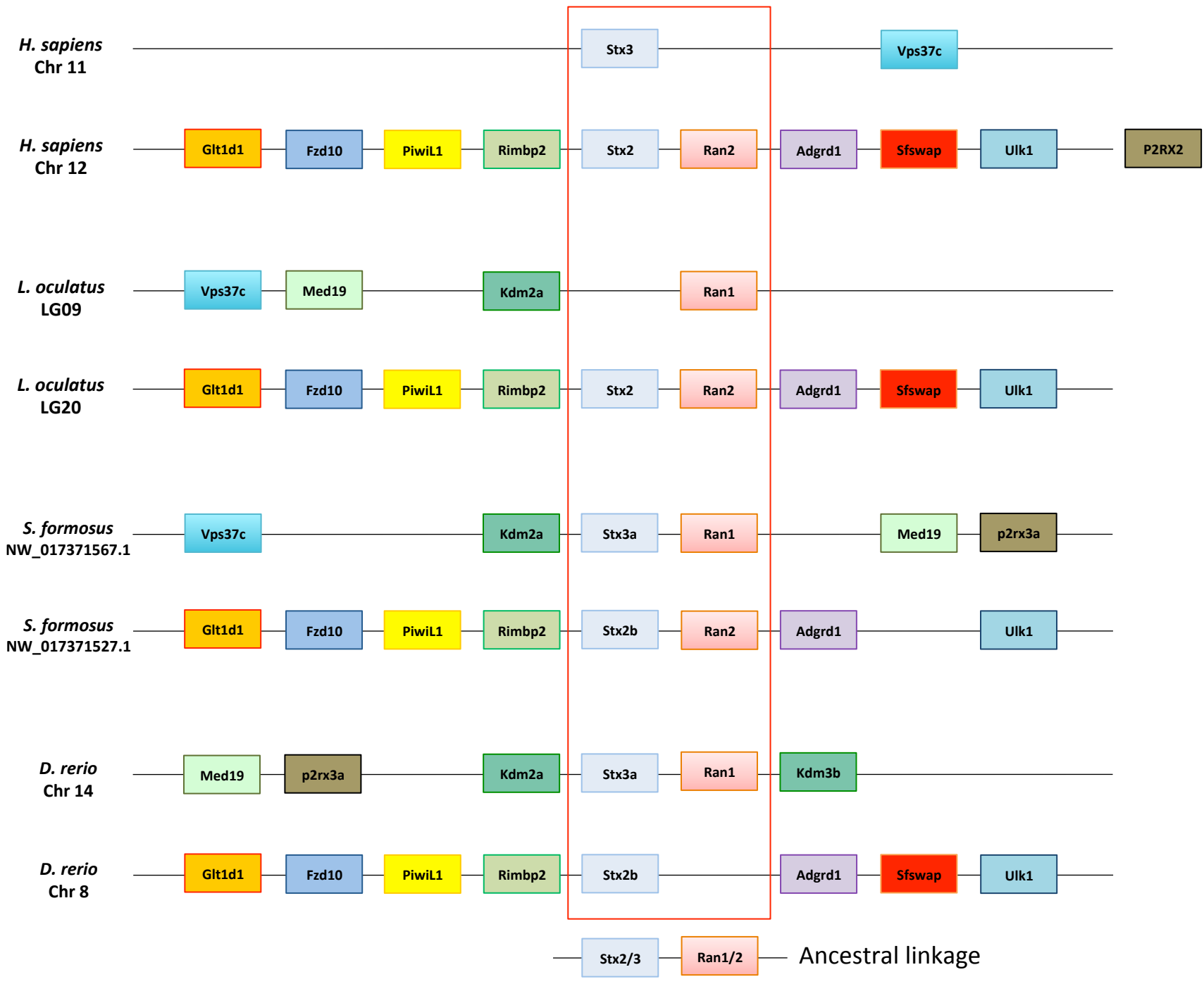

Supplement: S3 Fig — A color code has been used to represent orthologue genes. (PDF) [file pone.0196930.s003.pdf]

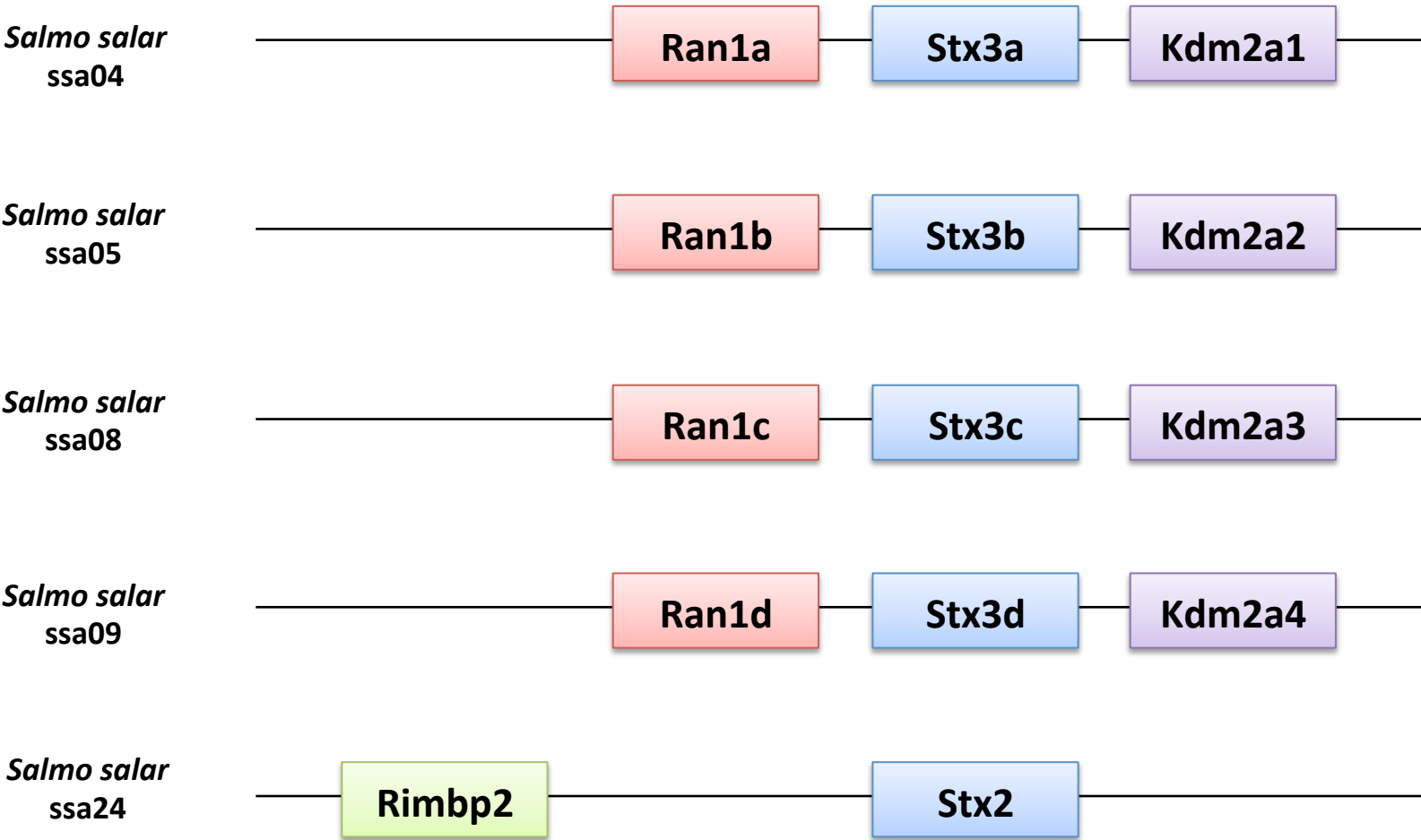

Supplement: S4 Fig — (PDF) [file pone.0196930.s004.pdf]

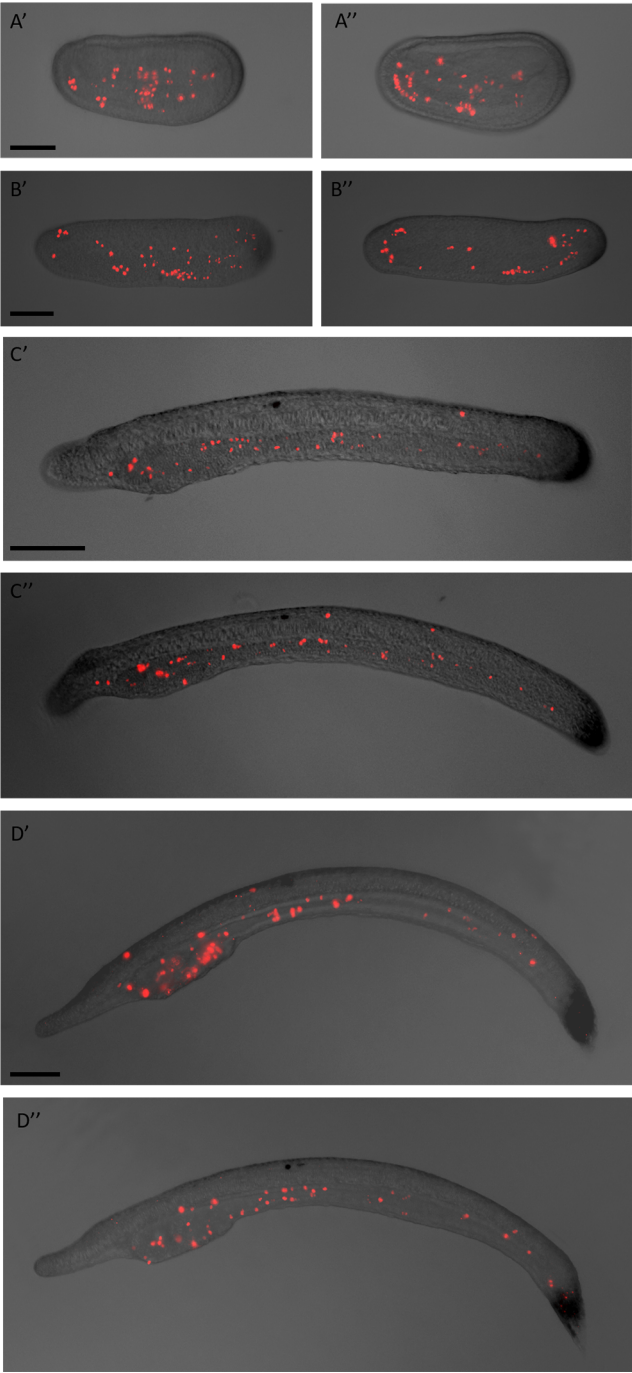

Supplement: S5 Fig — Collection of two series of embryos (A’-A”, B’-B”, C’-C”, D’-D” represent distinct specimens) showing slightly different PHH3 immunolocalization signals (red). Scale bars: 60 μm. Embryos orientation: anterior to the left, dorsal to the top. (PDF) [file pone.0196930.s005.pdf]
