# Supplementary material for: Ran GTPase, an eukaryotic gene novelty, is involved in amphioxus mitosis
Source: PLoS One. 2018 Oct 9;13(10):e0196930. doi: 10.1371/journal.pone.0196930 (PMC6177115; doi:10.1371/journal.pone.0196930)
Supplement: S1 File — (PDF) [file pone.0196930.s006.pdf]

>EYB33785.1_Ran_Fusarium_graminearum
MAEQQTPTFKLVLVGDGGTGKTTFVKRHLTGEFEKKYMATLGVEVHPLGFTTNFGQIQFDVWDTAGQE
KFGGLRDGYYINGQCGIIMFDVTSRITYKNVPNWHRDLVRVCENIPIVLCGNKVDVKERKVKAKTITF
HRKKNLQYYDISAKSNYNFEKPFLWLARKLVGNPQLEFVAAPALAPPTAQVDEKLLEEYRKEMDEAAA
MPLPGELSDDDL

>AJV74468.1_Gsp1p_Saccharomyces_cerevisiae
MSAPAANGEVPTFKLVLVGDGGTGKTTFVKRHLTGEFEKKYIATIGVEVHPLSFYTNFGEIKFDVWDT
AGQEKFGGLRDGYYINAQCAIIMFDVTSRITYKNVPNWHRDLVRVCENIPIVLCGNKVDVKERKVKAK
TVTFHRKKNLQYYDISAKSNYNFEKPFLWLARKLAGNPQLEFVASPALAPPEVQVDEQLMQQYQQEME
QATALPLPDEDDADL

>AJT75514.1_Gsp2p_Saccharomyces_cerevisiae
MSVPAQNNTEVPTFKLVLVGDGGTGKTTFVKRHLTGEFEKKYIATIGVEVHPLSFYTNFGEIKFDVWD
TAGQEKFGGLRDGYYINAQCAIIMFDVTSRITYKNVPNWHRDLVRVCENIPIVLCGNKVDVKERKVKA
KTITFHRKKNLQYYDISAKSNYNFEKPFLWLARKLAGNPQLEFVASPALAPPEVQVDEQLMHQYQQEM
DQATALPLPDEDDADL

>CAA66047.1_Ran1_Arabidopsis_thaliana
MALPNQQTVDYPSFKLVIVGDGGTGKTTFVKRHLTGEFEKKYEPTIGVEVHPLDFFTNCGKIRFYCWD
TAGQEKFGGLRDGYYIHGQCAIIMFDVTARLTYKNVPTWHRDLCRVCENIPIVLCGNKVDVKNRQVKA
KQVTFHRKKNLQYYEISAKSNYNFEKPFLYLARKLAGDQNLHFVETPALAPPEVHIDIADQQKNEAEL
LQAAAQPLPDDDDDIFE

>Q7F7I7.1_RAN1_Oryza_sativa
MALPNQQTVDYPSFKLVIVGDGGTGKTTFVKRHLTGEFEKKYEPTIGVEVHPLDFFTNCGKIRFYCWD
TAGQEKFGGLRDGYYIHGQCAIIMFDVTSRLTYKNVPTWHRDLCRVCENIPIVLCGNKVDVKNRQVKA
KQVTFHRKKNLQYYEVSAKSNYNFEKPFLYLARKLAGDGNLHFVETPALAPPDVTIDLAAQQQHEAEL
AAAAAQPLPDDDDDLIE

>KNE92840.1_Ran_Puccinia_striiformis
MAESTTVPTFKLVLVGDGGTGKTTFVKRHLTGEFEKKYIATLGVEVHPLGFHTNHGMICFNVWDTAGQ
EKFGGLRDGYYIQGQCGIIMFDVTSKITYKNVPNWFRDLERVCEGIPIVLCGNKVDVKERKVKTGSVT
FHRKKNLQYFEISAKSNYNFEKPFLWLARKLSGTTNLEFVAAPALAPPEVAVDANLMETYNRELEAAA
AAPLPDEDDADL

>OEU19320.1_Ran_Fragilariopsis_cylindrus
MADPATPEFKLILVGDGGVGKTTFVKRHLTGEFEKKYVATLGVEVHPLNFHTNRGPIKFNVWDTAGQE
KFGGLRDGYYIQGQCAIIMFDVTSRITYKNVPNWHRDLTRVCENIPIVLCGNKVEIKDRKVKAKQITF
HRKKNLQYYDISAKSNYNFEKPFLWLARKLSGDNALHFVEAPALQPPEFQFDENLKNQYEQELQAAAA
QPLPEDDDDDL

>XP_001749950.1_Ran_Monosiga_brevicollis
MEAPRFKLVLVGDGGTGKTTFVKRHLTGEFEKKYVATLGADVHPLSFQTNCGPIIFDVWDTAGQEKFG
GLRDGYYIHGKCAIIMFDVTARVTYKNVPNWHRDLVRVCENIPMVLCGNKVDMKDRKVKAKQITFHRK
KMLQYYDISAKSNFNFEKPFLWLARRLSGQPNLEFVAAPALRPAEVEIDHAAMQRAEEEMRMAQQAEL
PDDDDDDL

>XP_004344244.1_Ran_Capsaspora_owczarzaki
MENQDIPTFKLVLVGDGSTGKTTFVKRHISGEFERKYVATIGVEVHPLLFHTSFGAIKFNVWDTAGQE
KFGGLRDGYYIQGQCAVIMFDVTARVTYKNVPNWYRDLTRVCEHIPIVLCGNKVDVKDRKVKAKTITF
HRKKNLQYYDISAKSNYNFEKPFLWLAKKLIDPSLEFVSMPALKPVEATVDPALIQQYEAELEEARNT
AIPDADDDEDI

>EKC36560_Ran_Crassostrea_gigas
MADQIPTFKLVLVGDGGVGKTTFVKRHVTGEFEKKYVATLGVEVHPLKFHTTRGEIIFNVWDTAGQEK
FGGLRDGYYIQGQCAIIMFDVTSRVTYKNVPNWHRDLVRVCENIPIVLCGNKVDIKDRKVKAKAIVFH
RKKNLQYYDISAKSNYNFEKPFLWLARKLVGDANLEFVAMPALAPPDIKMDPNLAAQYEQELQAAVEV
ALPDDDDDL

>Ocbimv22034405_Ran_Octopus_bimaculoides
MCDKMPTFKLVLVGDGGVGKTTFVKRHLTGEFEKKYVATLGVEVHPLVFYTARGPIRFNVWDTAGQEK
FGGLRDGYYIQGQCAIIMFDVTSRVTYKNVPNWHRDLVRVCENTPIVLCGNKVDVKDRKVKAKAITFH
RKKNLQYYDISAKSNYNFEKPFLWLARKLIGDANLEFVEMPALQPPEVEMSQSLAQQYEKELEVAQEI
ALPDEDEDL

>XP_003385856.1_Ran_Amphimedon_queenslandica
MAAQQEPVATFKLVLVGDGGTGKTTFVKRHLTGEFEKKYVATLGVEVHPLVFYTNRGPIRFNVWDTAG
QEKFGGLRDGYYIQGQCAIIMFDVTSRVTYKNVPNWHRDLVRVCEGIPIVLCGNKVDIKDRKVKAKAI
TFHRKKNLQYYDISAKSNYNFEKPFLWLARKLVGDPNLEFVEMPALEPPEVQMDPTLAAKYEQELKSA
AEVALPDDNDDDDL

>XP_003388238.1_RanL_Amphimedon_queenslandica
MAAQQEPVATFKLVLIGDGCTGKTAFITRFLTGKFEKNYRATLGVEIHQLVFQTNKGPIQFNVWDTAG
LEKFGGLRDGYYILEQRAMAGIIMFDVTSRATYKNVPNWHRDLIRVCEDIPIVLCGNKVDIEDRKVKA
KTVTWHRKKNNLEYCDISVKCNHNLEKPFLLLARKLANDPNLEFVEMPAFEPPEVQMDPTLAAKYEAE
LKQREEAACSDNDDDSDD

>KXJ29072.1_Ran_Exaiptasia_pallida
MSAGPGVQPVATFKLVLVGDGGTGKTTFVKRHLTGEFEKKYVATLGVEVHPLVFYTNRGPIRFNVWDT
AGQEKFGGLRDGYYIQGQCAIIMFDVTSRVTYKNVPNWHRDLIRVCENIPIVLCGNKVDIKDRKVKAK
SITFHRKKNLQYYDISAKSNYNFEKPFLWLLRKLVGDSNLELVEMPALEPPEVTMDPSLAQKYEADLK
EAQNTALPEEDDEDI

>MLRB2002198_Ran_Mnemiopsis_leidyi
MAANTDAQKKDGAPVATFKLVLVGDGGTGKTTFVKRHLTGEFEKRYVTLGVEVHPLVFYTNFGPIFDV
WDTAGQEKFGGLRDGYYIGGQCAIIFFDVTSRVTYKNVPNWHRDLVRVLENVPIVLCGNKVDIKDRKV
KAKQITFHRKKNLQYYDISAKSNYNFEKPFLWLARKISGEAGLQFVEAPALAPPEVKVDPALMAQYEK
ELNQAANMALPDDENDDDDL

>XP_001629438.1_Ran_Nematostella_vectensis
MSQPVQPVATFKLVLVGDGGTGKTTFVKRHLTGEFEKKYIATLGVEVHPLIFFTSRGPIKFNVWDTAG
QEKFGGLRDGYYIQGQCAIIMFDVTSRVTYKNVPNWHRDLERVCENIPIVLCGNKVDIKDRKVKAKAI
TFHRKKNLQYYDLSAKSNYNFEKPFLWLARKLVGDPNLEFVEMPALQPPEVQMDPNMAKQYEQDLNDA
QNTALPDEDDEDL

>NP_499369.1_Ran_Caenorhabditis_elegans
MSGGDGIPTFKLVLVGDGGTGKTTFVKRHLTGEFEKKYVATLGVEVHPLVFHTNRGQIRFNVWDTAGQ
EKFGGLRDGYYIQGQCAIIMFDVTARVTYKNVPNWHRDLARVCENIPIVLCGNKVDVKDRKVKAKTIT
FHRKKNLQYYDISAKSNYNFEKPFLWLARKLLGDPNLEFVAMPALAPPEVQMDPAMIAEYEKDLDNAA
KADLPDDDDDL

>XP_013777947.1_Ran_Limulus_polyphemus
MQQNQTDLGDGIPTFKCVLVGDGGTGKTTFVKRHLTGEFEKKYVATLGVEVHPLLFHTNRGPVRFNVW
DTAGQEKFGGLRDGYYIQGQCAVIMFDVTSRVTYKNVPNWHRDLVRVCENIPIVLCGNKVDIKDRKVK
AKSIVFHRKKNLQYYDISAKSNYNFEKPFLWLARKLIGDPNLEFVAMPALAPPEVAMDPEWQAKLENE
MKEAQNTSLPDDDDDDL

>JAN90411.1_Ran_Daphnia_magna
MAAEQDIPTFKCVLVGDGGTGKTTFVKRHMTGEFEKKYVATLGVEVHPLVFHTNRGAIRFNVWDTAGQ
EKFGGLRDGYYIQGQCAIVMFDVTSRITYKNVPNWHRDLVRVCENIPIVLCGNKVDIKDRKVKAKSIV
FHRKKNLQYYDISAKSNYNFEKPFLWLARKLVGDPNLEFVAMPALLPPEVKMDKAWQDQLEREMEEAS
KTALPEDDEDL

>AAF30287.1_Ran_Drosophila_melanogaster
MAQEGQDIPTFKCVLVGDGGTGKTTFVKRHMTGEFEKKYVATLGVEVHPLIFHTNRGAIRFNVWDTAG
QEKFGGLRDGYYIQGQCAVIMVDGNSRVTYKNVPNWHRDLVRVCENIPIVLCGNKVDIKDRKVKAKSI
VFHRKKNLQYYDISAKSNYNFEKPFLCWARKLVGDPNLEFVAMPALLPPEVKMDKDWQAQIERDLQEA
QATALPDEDEEL

>ADI33946.1_RanL_Drosophila_melanogaster
MQPQEEVKAIFKLILIGDGGTGKTTLVKRHLTGEFKMQYNATLGVEVEQLLFNTNRGVFRIDVWDTAG
QERYGGLRDGYFVQAQCAIIMFDVASSNTYNNVKRWHRDLVRVCGNIPIVICGNKVDIMHKKTWKKGV
DFDRKTNIYLIEMSAKSNYNVEKPFVYLLRKLVGDPSLQLVQSPAIQPPKVVFTDEMSRQVESLFNEA
KSKPLPPIYDIDL

>XP_009051818.1_Ran_Lottia_gigantea
FQLVLVGDGGVGKTTFVKRHVTGEFEKKYVATLGVEVNSMLFNTNRGHIKFNVWDTAGQEKFGGLRDG
YYIQGQCAIIMFDVTSRITYKNVPNWHRDLVRVCENIPIVLCGNKVDIKDRKVKAKSIVFHRKKNLQY
YDISAKSNYNFEKPFLWLAKKLVGDADLTFVEMPALKPPEVQMDEATLRRYEEELRDAAETALPDDDD
NDL

>XP_006821108.1_Ran_Saccoglossus_kowalevskii
MAQQNAGDEVPTFKLVLVGDGGTGKTTFVKRHLTGEFEKKYVATLGVEVHPLLFHTNRGPIRFNVWDT
AGQEKFGGLRDGYYIQGQCAIIMFDVTSRVTYKNVPNWHRDLVRVCENIPIVLCGNKVDIKDRKVKAK
SITFHRKKNLQYYDISAKSNYNFEKPFLWLARKLSGDPNLEFVAMPALAPPEVNMDPQLAAKYEEDLK
HAQATALPDEDDDLL

>ELT90460.1_Ran_Capitella_teleta
MAQPDPNIATFKLVLVGDGGVGKTTFVKRHLTGEFEKKYIATLGVEVHPLVFHTNRGAIRFNVWDTAG
QEKFGGLRDGYYIQGNCAIIMFDVTARITYKNVPNWHRDLVRVCETIPIVLCGNKVDIKDRKVKAKSI
VFHRKKNLQYYDISAKSNYNFEKPFLWLARKLTGEPNLEFVAMPALAPPEVQMDAAMAAKYEEELKVA
QTTALPEEEDDL

>XP_014677940.1_Ran_Priapulus_caudatus
MSTGADDIPTFKCVLVGDGGTGKTTFVKRHLTGEFEKKYVATLGVEVHPLVFHTNRGPIRFNVWDTAG
QEKFGGLRDGYYIQGQCAIIMFDVTSRVTYKNVPNWHRDLVRVCENIPIVLTGNKVDIKDRKVKAKSI
VFHRKKNLQYYDISAKSNYNFEKPFLWLARKLIGDPNLEFVAMPALAPPEVQMDPEQVKQYEEELKLA
QQSTLPDDDDD

>CCD74879.1_Ran_Schistosoma_mansoni
MADQPPVAAFKLVLVGDGGTGKTTFVKRHITGEFEKKYVATLGVEVHPLDFHTTRGQEKFGGLRDGYY
IQGRCAIIMFDVTSRVTYKNVPNWHRDLVRVCENIPIVLCGNKVDIQDRKVKAKSITFHRKKNLQYYD
ISAKSNYNFEKPFLWLFRKLVGDPNLEFVEMPAMQPPEIQIDPYLVRQYEQEIQMAAEAPLPDEGDED
L

>XP_013392349.1_Ran_Lingula_anatina
MATQQQGTDEPTFKLVLVGDGGVGKTTFVKRHLTGEFEKKYVATLGVEVHPLLFHTNRGPIKFNVWDT
AGQEKFGGLRDGYYIQGQCAIIMFDVTARVTYKNVPNWHRDLVRVCENIPMVLCGNKVDIKDRKVKAK
AIVFHRKKNIQYYDISAKSNYNFEKPFLYLARKLVGDPNLEFVAMPALQPPEVTMDPSIAKQYERELE
MAQATALPEDDEDI

>XP_011676375.1_Ran_Strongylocentrotus_purpuratus
MCIAYKEFSSQREPVSYRKMNADSITPGVTPNDPIATFKLVLVGDGGTGKTTFVKRHLTGEFEKRYLA
TLGVEVHPLVFYTNRGPIRFNVWDTAGQEKFGGLRDGYYIQGQCAIIMFDVTSRVTYKNVPNWHRDLV
RVCESIPIVLCGNKVDIKERKVKAKTITFHRKKNLQYYDISAKSNYNFEKPFLWLARKLCGDAEMNFV
EMPALKPPEVHMDPNLQKQYETELQEAQNTALPEDEDDI

>XP_002612124.1_Ran_Branchiostoma_floridae
MAQEIAGANDRQPATFKLVLVGDGGTGKTTFVKRHVTGEFEKKYVATLGVEVHPIKFNTNRGEIKFNV
WDTAGQEKFGGLRDGYYIQGQCAIIMFDVTSRVTYKNVPNWHRDLVRVCENIPIVLCGNKVDIKDRKV
KAKAITFHRKKNLQYYDISAKSNYNFEKPFLWLARKLAGDANLEFVEMPALQPPEVQMDPELASQYER
DLKQAQETALPEEDDDL

>Sc0000461_Ran_Branchiostoma_lanceolatum
ANDRQPATFKLVLVGDGGTGKTTFVKRHVTGEFEKKYVATLGVEVHPIKFNTNRGEIKFNVWDTAGQE
KFGGLRDGYYIQGQCAIIMFDVTSRVTYKNVPNWHRDLVRVCENIPIVLCGNKVDIKDRKVKAKAITF
HRKKNLQYYDISAKSNYNFEKPFLWLARKLAGDANLEFVEMPALQPPEVQMDPELASQYERDLK

>XP_002128496.1_Ran_Ciona_robusta
MQQQQQDDLVVKLVLVGDGGVGKTTFVKRHLTGEFEKKYVATLGVEVHPIVFQTQRGRIRFNVWDTAG
QEKFGGLRDGYYIQGQCAIIMFDVTSRVTYKNVPNWHKDLVRVCEDIPIVLVGNKVDIKDRKVKAKAI
NFHRKKNLQYYDISAKSNYNFEKPFLWMARKLMGDPNLEFCAMPAMLPPEVKLDDNLMKQYEEELQNA
QQCSLPDEDDDL

>CBY09463.1_Ran_Oikopleura_dioica
MAAADQEMPTFKLVLVGDGGVGKTTFVKRHLTGEFEKKYVATLGVEVHPLVFHTNRGPLRFNVWDTAG
QEKFGGLRDGYYIQGQCAIVMFDVTSRVTYKNVPNWHRDLTRVCENIPIVLTGNKVDIKDRKVKAKSI
VFHRKKNLQYYDISAKSNYNFEKPFLWLARKLVGDPNLEFVAAPALAPPEVQMDPQMIAAYEDELKKA
SESALPDEDDDL

>JL10651_Ran_Lethenteron_japonicum
LFFVYHYGWFCQQLVLVGDGGTGKTTFVKRHLTGEFEKKYVATLGVEVHPLVFHTNRGAIKFNVWDTA
GQEKFGGLRDGYYIQGQCAIIMFDVTSRVTYKNVPNWHRDLVRVCENIPIVLCGNKVDIKDRKVKAKA
IVFHRKKNLQYYDISAKSNYNFEKPFLWLARKLIGDPNLEFVAMPALAPPEVQMDPQLVAQYEEDLKV
AQATALPDEEDDL

>XP_007906018.1_Ran_Callorhinchus_milii
MGDQGEPQVQFKLVLVGDGGTGKTTFVKRHLTGEFEKKYVATLGVEVHPLMFHTNRGAIKFNVWDTAG
QEKFGGLRDGYYIQAQCAIIMFDVTSRVTYKNVPNWHRDLVRVCENIPIVLCGNKVDIKDRKVKAKSI
VFHRKKNLQYYDISAKSNYNFEKPFLWLARKLIGDPNLEFVAMPALAPPEVQMDPALAAQYEEELKVA
QSTALPDEDDDL

>ENSLACP00000021390.1_Ran2_Latimeria_chalumnae
MASPPDVQFKLVLVGDGGTGKTTFVKRHLTGEFEKKYVATLGVEVHPLVFHTNRGSVKFNVWDTAGQE
KFGGLRDGYYIQAQCAIIMFDVTSRVTYKNVPNWHRDLVRVCENIPIVLCGNKVDIKDRKVKAKSIVF
HRKKNLQVCDR

>XP_006640309.1_Ran2_Lepisosteus_oculatus
MATQGEPQVQFKLVLVGDGGTGKTTFVKRHLTGEFEKKYVATLGVEVHPLVFHTNRGAIKFNVWDTAG
QEKFGGLRDGYYIQAQCAIIMFDVTSRVTYKNVPNWHRDLVRVCENIPIVLCGNKVDIKDRKVKAKSI
VFHRKKNLQYYDISAKSNYNFEKPFLWLARKLIGDPNLEFVAMPALAPPEVVMDPALAAQYEQDLQVA
QSTALPDDEDDL

>ENSLOCP00000010056.1_Ran1_Lepisosteus_oculatus

ATLGVEVHPLVFHTNRGAIKFNVWDTAGQEKFGGLRDGYYIQAQCAIIMFDVTSRVTYKNVPNWHRDL
VRVCENIPIVLCGNKVDIKDRKVKAKSIVFHRKKNLQYYDISAKSNYNFEKPFLWLARKLIGDPNLEF
VAMPALAPPEVSMDPSLAAQYEHDLKVASETALPDEEDDL

>NP_990589.1_Ran_Gallus_gallus
MAAQGEPQVQFKLVLVGDGGTGKTTFVKRHLTGEFEKKYVATLGVEVHPLVFHTNRGPIKFNVWDTAG
QEKFGGLRDGYYIQAQCAIIMFDVTSRVTYKNVPNWHRDLVRVCENIPIVLCGNKVDIKDRKVKAKSI
VFHRKKNLQYYDISAKSNYNFEKPFLWLARKLIGDPNLEFVAMPALAPPEVVMDPALAAQYEQDLQIA
QTTALPDEDDDL

>ENSXETP00000049065.3_Ran_Xenopus_tropicalis
MAAQGEPQVQFKLVLVGDGGTGKTTFVKRHLTGEFEKKYVAIATLGVEVHPLVFHTNRGPIKFNVWDT
AGQEKFGGLRDGYYIQAQCAIIMFDVTSRVTYKNVPNWHRDLVRVCENIPIVLCGNKVDIKDRKVKAK
SIVFHRKKNLQYYDISAKSNYNFEKPFLWLARKLIGDPNLEFVAMPALAPPEVVMDPALAAQYEQDLQ
HAQATALPDEDDDL

>XP_008117245.1_Ran_Anolis_carolinensis
MAAPQGEPQVQFKLVLVGDGGTGKTTFVKRHLTGEFEKKYVATLGVEVHPLVFHTNRGPIKFNVWDTA
GQEKFGGLRDGYYIQAQCAIIMFDVTSRVTYKNVPNWHRDLVRVCENIPIVLCGNKVDIKDRKVKAKS
IVFHRKKNLQYYDISAKSNYNFEKPFLWLARKLIGDPNLEFVAMPALAPPEVVMDPALAAQYEQDLQI
AQTTALPDEDDDL

>NP_006316.1_RAN_Homo_sapiens
MAAQGEPQVQFKLVLVGDGGTGKTTFVKRHLTGEFEKKYVATLGVEVHPLVFHTNRGPIKFNVWDTAG
QEKFGGLRDGYYIQAQCAIIMFDVTSRVTYKNVPNWHRDLVRVCENIPIVLCGNKVDIKDRKVKAKSI
VFHRKKNLQYYDISAKSNYNFEKPFLWLARKLIGDPNLEFVAMPALAPPEVVMDPALAAQYEHDLEVA
QTTALPDEDDDL

>NP_571384.1_Ran_Danio_rerio
MAENEPQVQFKLVLVGDGGTGKTTFVKRHLTGEFEKKYVATLGVEVHPLVFHTNRGAIKYNVWDTAGQ
EKFGGLRDGYYIQAQCAIIMFDVTSRVTYKNVPNWHRDLVRVCENIPIVLCGNKVDIKDRKVKAKSIV
FHRKKNLQYYDISAKSNYNFEKPFLWLARKLIGDPNLEFVEMPALAPPEIAMDPTLAAQYEHDLKVAS
ETALPDEDDDL

>XP_018593764.1_Ran1_Scleropages_formosus
MAEGEPQVQFKLVLVGDGGTGKTTFVKRHLTGEFEKKYVATLGVEVHPLVFHTNRGAIKFNVWDTAGQ
EKFGGLRDGYYIQAQCAIIMFDVTSRVTYKNVPNWHRDLVRVCENIPIVLCGNKVDIKDRKVKAKSIV
FHRKKNLQYYDISAKSNYNFEKPFLWLARKLIGDPNLEFVAMPALAPPEVAMDPSLAAQYEHDLKE

>XP_018587028.1_Ran2_Scleropages_formosus
MAMQAEPQVQFKLVLVGDGGTGKTTFVKRHLTGEFEKKYVATLGVEVHPLMFHTNRGAIKFNVWDTAG
QEKFGGLRDGYYIQAQCAIIMFDVTSRVTYKNVPNWHRDLVRVCENIPIVLCGNKVDIKDRKVKAKAI
VFHRKKNLQYYDISAKSNYNFEKPFLWLARKLIGDPNLEFVAMPALAPPEVVMDPQLAKQYEEDLQVA
QSTALPDDEDDL

>NP_001135061.1_Ran1a_Salmo_salar
MAEPDVQFKLVLCGDGGTGKTTFVKRHLTGEFEKKYVATLGVEVHPLVFHTTRGTIKYNVWDTAGQEK
FGGLRDGYYIQAQCAIIMFDVTSRVTYKNVPNWHRDLVRVCENIPIVLCGNKVDIKDRKVKAKSIVFH
RKKNLQYYDISAKSNYNFEKPFLWLARKLVGDPNLEFVEMPALAPPEVVMDATLAAQYENDLKVAAET
ALPDEDDDL

>CAA10191.1_Ran1b_Salmo_salar
MAEGEPQVQFKLVLVGDGGTGKTTFVKRHLTGEFEKKYVATLGVEVHPLVFHTNRGAIKYNVWDTAGQ
EKFGGLRDGYYIQAQCAIIMFDVTSRVTYKNVPNWHRDLVRVCENIPIVLCGNKVDIKDRKVKAKSIV
FHRKKNLQYYDISAKSNYNF

>XP_014064423.1_Ran1c_Salmo_salar
MAEPDVQFKLVLCGDGGTGKTTFVKRHLTGEFEKKYVATLGVEVHPLVFHTTRGTIKYNVWDTAGQEK
FGGLRDGYYIQAQCAIIMFDVTSRVTYKNVPNWHRDLVRVCENIPIVLCGNKVDIKDRKVKAKSIVFH
RKKNLQYYDISAKSNYNFEKPFLWLARKLVGDPNLEFVEMPALAPPEVVMDASLAAQYENDLKVAAET
ALPDEDDDL

>NP_001117103.1_Ran1d_Salmo_salar
MAEGEPQVQFKLVLVGDGGTGKTTFVKRHLTGEFEKKYVATLGVEVHPLVFHTNRGAIKYNVWDTAGQ
EKFGGLRDGYYIQAQCAIIMFDVTSRVTYKNVPNWHRDLVRVCENIPIVLCGNKVDIKDRKVKAKSIV
FHRKKNLQYYDISAKSNYNFEKPFLWLARKLIGDPNLEFVAMPALAPPEILMDPSLAAQYEHDLKVAS
ETALPDEDDDL
