# Supplementary material for: Ran GTPase, an eukaryotic gene novelty, is involved in amphioxus mitosis
Source: PLoS One. 2018 Oct 9;13(10):e0196930. doi: 10.1371/journal.pone.0196930 (PMC6177115; doi:10.1371/journal.pone.0196930)
Supplement: S2 File — (PDF) [file pone.0196930.s007.pdf]

>XP_009066890.1_Stx7/12_Lottia_gigantea
MASGQGRYGSNNYFDDPGYREYGGGFSGGSASVSGTIEQIKSNIFKINSGANSIDKAMKSIGTDRDSV
QLRDKIHETSQNISKLVQDSMRQLKVVGNKPADKQQKIQLSKLRNDFQESVERFQGLQKMATEKVKSS
VKLGSRKATPQVDSGMTGWNDPDDFSDKTQFYHQEERRAELQEQQQVVEDDLALIREREERIRQLESD
ILDVNEIFRDLGALVSEQGETLDTIEANVERTYGNVEQANSQLSQAAVYQKKARKKKCCLLVILLVVA
IVITIIIVVSVKS

>XP_009047532.1_Stx17_Lottia_gigantea
MASFERNQRSIEFTTAQKYPIRRFEVSIKKFVKVLTIDLERLDKHRVNIDRLTHQENWTLLNKEQVNA
SRTVQQIKANVREIEKARGQIIEDDLHQFDLCIEEVKLKAIQAVKDFLVLSQGGGSSPLGELTLTPGS
ESPPPTSISTDYTPTPACDIPIRSSGISPSGSTSFSLPSSSQMTQIQLYTNPPASTVNPETLASWEHL
QEDLIDLNGMINDFATMVEEQGEKVDKIEDNIEKAELDV

>XP_001633611.1_Stx16_Nematostella_vectensis
MAVRSLTDVYFNLRTEISRFKAYSMGDSPVDDDTVALVQRVDPELGTQAPISSLPPQWIDAVEEIQYE
ITRIKQRMKDLSTLHDRHLNRPTLDDSIDEEQTIEITTKEITQMFHQCQNAVQKMSRQSRTAGKQEQR
LLKNVISSLAVSLQELSTNFRKSQSTYLKRLKNREERERQFFDTGLPSTSSALMNEDVVEDDDLYDRG
FTNDQMRLVEDNSAIVEQREKEIQSIVQSISELNEIFRDLATMIVEQGSILDRIDYNVEQASVKVEQG
LEQLKKAEQHQKSSRKMLCIILISVVLIILLVALVITKS

>XP_001628196.1_Stx7/12L_Nematostella_vectensis
MSRGEFGRGDGRDYGAFGDGRSDLHGQGQSSHRAGYQREGNAREEFQNLSDSVSSSIFQINNNTSALE
RILRQITSGKDKVSAEKIHRIQQGTNKLASETTHLLKQMSTMCGGTSPSSRQQRIQHERLKEEFRDSI
SRYYSVQNKVAEQEKLIVRSTREPGYSQLDDDFGTEKSSLIEEDSRRASQEQLSEQITIDEGLIYERE
ERIRQIEGDILDINEIFRDLATMVYEQGETIDSIEGNIEQAYNNVGSANIQLQKASKLQKAARKKMCL
LLLVLVIIGGIIALIVVLT

>XP_001636651.1_Stx7/12_Nematostella_vectensis
IEERERAIRQLEADIVGVNEIFRDLGNMIHEQGEVIDSIEANVETAAVHVETANVQLDKARGYQVKNP
ERPSSFL

>ENSP00000378585.3_STX1A_Homo_sapiens
MKDRTQELRTAKDSDDDDDVAVTVDRDRFMDEFFEQVEEIRGFIDKIAENVEEVKRKHSAILASPNPD
EKTKEELEELMSDIKKTANKVRSKLKSIEQSIEQEEGLNRSSADLRIRKTQHSTLSRKFVEVMSEYNA
TQSDYRERCKGRIQRQLEITGRTTTSEELEDMLESGNPAIFASGIIMDSSISKQALSEIETRHSEIIK
LENSIRELHDMFMDMAMLVESQTMWRGPCLTPRRPSSTRARRAGRKS

>ENSP00000215095.5_STX1B_Homo_sapiens
MKDRTQELRSAKDSDDEEEVVHVDRDHFMDEFFEQVEEIRGCIEKLSEDVEQVKKQHSAILAAPNPDE
KTKQELEDLTADIKKTANKVRSKLKAIEQSIEQEEGLNRSSADLRIRKTQHSTLSRKFVEVMTEYNAT
QSKYRDRCKDRIQRQLEITGRTTTNEELEDMLESGKLAIFTDDIKMDSQMTKQALNEIETRHNEIIKL
ETSIRELHDMFVDMAMLVESQGEMIDRIEYNVEHSVDYVERAVSDTKKAVKYQSKARRKKIMIIICCV
VLGVVLASSIGGTLG

>ENSP00000261653.6_STX2_Homo_sapiens
MRDRLPDLTACRKNDDGDTVVVVEKDHFMDDFFHQVEEIRNSIDKITQYVEEVKKNHSIILSAPNPEG
KIKEELEDLNKEIKKTANKIRAKLKAIEQSFDQDESGNRTSVDLRIRRTQHSVLSRKFVEAMAEYNEA
QTLFRERSKGRIQRQLEITGRTTTDDELEEMLESGKPSIFTSDIISDSQITRQALNEIESRHKDIMKL
ETSIRELHEMFMDMAMFVETQGEMINNIERNVMNATDYVEHAKEETKKAIKYQSKARRKLMFIIICVI
VLLVILGIILATTLS

>ENSP00000300150.7_STX3_Homo_sapiens
MDEFFSEIEETRLNIDKISEHVEEAKKLYSIILSAPIPEPKTKDDLEQLTTEIKKRANNVRNKLKSME
KHIEEDEVRSSADLRIRKSQHSVLSRKFVEVMTKYNEAQVDFRERSKGRIQRQLEITGKKTTDEELEE

MLESGNPAIFTSGIIDSQISKQALSEIEGRHKDIVRLESSIKELHDMFMDIAMLVENQGEMLDNIELN
VMHTVDHVEKARDETKKAVKYQSQARKKLIIIIVLVVVLLGILALIIGLSVGLN

>ENSP00000378447.1_STX4_Homo_sapiens
MGMTARTKRTRSGSRWWCTRARHGWGARTRSSSTSPLGHPPQVRTIRQTIVKLGNKVQELEKQQVTIL
ATPLPEESMKQELQNLRDEIKQLGREIRLQLKAIEPQKEEADENYNSVNTRMRKTQHGVLSQQFVELI
NKCNSMQSEYREKNVERIRRQLKITNAGMVSDEELEQMLDSGQSEVFVSNILKDTQVTRQALNEISAR
HSEIQQLERSIRELHDIFTFLATEVEMQGEMINRIEKNILSSADYVERGQEHVKTALENQKKARKKKV
LIAICVSITVVLLAVIIGVTVVG

>ENSP00000367129.4_STX5_Homo_sapiens
MIPRKRYGSKNTDQGVYLGLSKTQVLSPATAGSSSSDIAPLPPPVTLVPPPPDTMSCRDRTQEFLSAC
KSLQTRQNGIQTNKPALRAVRQRSEFTLMAKRIGKDLSNTFAKLEKLTILAKRKSLFDDKAVEIEELT
YIIKQDINSLNKQIAQLQDFVRAKGSQSGRHLQTHSNTIVVSLQSKLASMSNDFKSVLEVRTENLKQQ
RSRREQFSRAPVSALPLAPNHLGGGAVVLGAESHASKDVAIDMMDSRTSQQLQLIDEQDSYIQSRADT
MQNIESTIVELGSIFQQLAHMVKEQEETIQSVLLFPLLPALSPGSTRTC

>ENSP00000440188.1_STX6_Homo_sapiens
MKDQMSTSSVQALAERKNRQALLGDSGSQNWSTGTTDKYGRLDRELQRANSHFIEEQQAQQQLIVEQQ
DEQLELVSGSIGVLKNMSQRIGGELEEQAVMLEDFSHELESTQSRLDNVMKKLAKVSHMTSDRRQWCA
IAILFAVLLVVLILFLVL

>ENSP00000356918.1_STX7_Homo_sapiens
MSYTPGVGGDPAQLAQRISSNIQKITQCSVEIQRTLNQLGTPQDSPELRQQLQQKQQYTNQLAKETDK
YIKEFGSLPTTPSEQRQRKIQKDRLVAEFTTSLTNFQKVQRQAAEREKEFVARVRASSRVSGSFPEDS
SKERNLVSWESQTQPQVQVQDEEITEDDLRLIHERESSIRQLEADIMDINEIFKDLGMMIHEQGDVID
SIEANVENAEVHVQQANQQLSRAADYQRKSRKTLCIIILILVIGVAIISLIIWGLNH

>ENSP00000305255.2_STX8_Homo_sapiens
MAPDPWFSTYDSTCQIAQEIAEKIQQRNQYERKGEKAPKLTVTIRALLQNLKEKIALLKDLLLRAVST
HQITQLEGDRRQNLLDDLVTRERLLLASFKNEGAEPDLIRSSLMSEEAKRGAPNPWLFEEPEETRGLG
FDEIRQQQQKIIQEQDAGLDALSSIISRQKQMGQEIGNELDEQNEIIDDLANLVENTDEKLRNETRRV
NMVDRKSASCGMIMVILLLLVAIVVVAVWPTN

>ENSP00000339350.4_STX10_Homo_sapiens
MSLEDPFFVVRGEVQKAVNTARGLYQRWCELLQESAAVGREELDWTTNELRNGLRSIEWDLEDLEETI
GIVEANPGKPAAQKSPSDLLDASAVSATSRYIEEQQATQQLIMDEQDQQLEMVSGSIQVLKHMSGRVG
EELDEQGIMLDAFAQEMDHTQSRMDGVLRKLAKVSHMTSDRRQWCAIAVLVGVLLLVLILLFSL

>ENSP00000363054.4_STX12_Homo_sapiens
MSYGPLDMYRNPGPSGPQLRDFSSIIQTCSGNIQRISQATAQIKNLMSQLGTKQDSSKLQENLQQLQH
STNQLAKETNELLKELGSLPLPLSTSEQRQQRLQKERLMNDFSAALNNFQAVQRRVSEKEKESIARAR
AGSRLSAEERQREEQLVSFDSHEEWNQMQSQEDEVAITEQDLELIKERETAIRQLEADILDVNQIFKD
LAMMIHDQGDLIDSIEANVESSEVHVERATEQLQRAAYYQKKSRKKMCILVLVLSVIILILGLIIWLV
YKTK

>ENSP00000354445.4_STX16_Homo_sapiens
MALVSGISLDPEAAIGVTKRPPPKWVDGVDEIQYDVGRIKQKMKELASLHDKHLNRPTLDDSSEEEHA
IEITTQEITQLFHRCQRAVQALPSRARACSEQEGRLLGNVVASLAQALQELSTSFRHAQSGYLKRMKN
REERSQHFFDTSVPLMDDGDDNTLYHRGFTEDQLVLVEQNTLMVEEREREIRQIVQSISDLNEIFRDL
GAMIVEQGTVLDRIDYNVEQSCIKTEDGLKQLHKAEQYQKKNRKMLVILILFVIIIVLIVVLVGVKSR

>ENSP00000259400.6_STX17_Homo_sapiens
MSEDEEKVKLRRLEPAIQKFIKIVIPTDLERLRKHQINIEKYQRCRIWDKLHEEHINAGRTVQQLRSN

IREIEKLCLKVRKDDLVLLKRMIDPVKEEASAATAEFLQLHLESVEELKKQFNDEETLLQPPLTRSMT
VGGAFHTTEAEASSQSLTQIYALPEIPQDQNAAESWETLEADLIELSQLVTDFSLLVNSQQEKIDSIA
DHVNSAAVNVEEGTKNLGKAAKYKLAALPVAGALIGGMVGGPIGLLAGFKVAGIAAALGGGVLGFTGG
KLIQRKKQKMMEKLTSSCPDLPSQTDKKCS

>ENSP00000426648.1_STX18_Homo_sapiens
MAVDITLLFRASVKTVKTRNKALGVAVGGGVDGSRDELFRRSPRPKGDFSSRAREVISHIGKLRDFLL
EHRKDYINAYSHTMSEYGRMTDTERDQIDQDAQIFMRTCSEAIQQLRTEAHKEIHSQQVKEHRTAVLD
FIEDYLKRVCKLYSEQRAIRVKRVVDKKRLSKLEPEPNTKTRESTSSEKVSQSPSKDSEENPATEERP
EKILAETQPELGTWGDGKGEDELSPEEIQMFEQENQRLIGEMNSLFDEVRQIEGRVVEISRLQEIFTE
KVLQQEAEIDSIHQLVVGATENIKEGNEDIREVPHL

>ENSP00000320679.2_STX19_Homo_sapiens
MKDRLQELKQRTKEIELSRDSHVSTTETEEQGVFLQQAVIYEREPVAERHLHEIQKLQESINNLADNV
QKFGQQQKSLVASMRRFSLLKRESTITKEIKIQAEYINRSLNDLVKEVKKSEVENGPSSVVTRILKSQ
HAAMFRHFQQIMFIYNDTIAAKQEKCKTFILRQLEVAGKEMSEEDVNDMLHQGKWEVFNESLLTEINI
TKAQLSEIEQRHKELVNLENQIKDLRDLFIQISLLVEEQGESINNIEMTVNSTKEYVNNTKEKFGLAV
KYKKRNPCRVLCCWCCPCCSSK

>Sc0000461:47545-58615_Stx1_Branchiostoma_lanceolatum
MTDYNTTQTDYRERCKGRIQRQLEITGKQTTNEELEEMLESGNPAIFTSGIIMDTQHAKQALSDIEAR
HNDIMKLESSIRELHDMFMDMAMLVEQQGEMIDRIEYNVEHAVDYVETAVGDTKKAVKYQSKARRKKI
MIIVCCAVLIVIIVGVFGGVFG

>Sc0000061:1161158-1167876_Stx7/12_Branchiostoma_lanceolatum
MVNQLGTAQDTHELRDKLHQMQHYTNQLAKDTNKYLKDLSNLPNPSSQSEQRQRKMQRERLTNDFSTA
LNNFQTVQRRAAEKERESVSRARANSGLPPGGSSMTAQMMEEESNLEMIRERETNIRQLEADIMDVNS
IFKDLATMVHEQGEMIDSIEANVENAAIHVESGNQQLRQASDYQKKSRRKLCILLIVLLIVGAVVALI
LYFTLKK

>Sc0000127:565408-573337_Stx16_Branchiostoma_lanceolatum
MATRSLTEIFILMRNNAAQNRHMFAEQVSTRSADDVSVPDDRMALVSGISTDPDASIGVHKSSFPPDW
GVEDVSYEITKIRQKRQTNEQTHAGRLHGGGTRHRDYHSGNHSDVSQMSTGHPEHREQESVRVQPGAE
GDQEHHVVSRQQPSGPLLTVQEGTVCVD

>Sc0000031:1448813-1473784_Stx5_Branchiostoma_lanceolatum
MFHKFPQISSRLLLCSPVYWCRVPSTCTGSRTGAAEDSRKFRVQLDQHCTVCLTELSLRHDLQTFSMT
TRRRRFDSGGSSNGNGGERTSVSSSLTYVVPSADMSCRDRTTEFISAVKSMQMRQGAGLNRPVSRDLR
QRSDFSHRAKRIGRDIANTFAKLEKLTILAKRKSLFDDKPDIASLNKQIAELQEFARSRGRQNGRHVQ
SHSNSVVVALQSKLATMSNDFKSVLEVRTENLKHQRSRREQFSQGPVSSSMPPSTYNAPNGVHPVTRR
DEGYPVRRGGHRHRCTGEAETPETAATRRGG

>Sc0000156:436776-445394_Stx6/10_Branchiostoma_lanceolatum
MSLEDPFFVVKDEVQKAVQNATGLYQRWCELLEDPVSVSKEEYDWTSNELRNSLRSIEWDLEDLDETI
NILCRSNPRKFKIDQQELGDRRAFISRTRQSVKEMKEHMASPSAKARIEGRNRQHLFNGPSKRQDRYT
KLDSEMENTNQKFIGETRQQQ

>Sc0000045:708115-724151_Stx8_Branchiostoma_lanceolatum
MLDNLASKEKQLNDASKNDQGPTCLVTKGQQVSMTTPGLPRSRRKPVGWACRTSASSRQELWTQDQGL
DVLSTIIARQKQLGQAIGDEVDLQKS

>Sc0000014:993112-998638_Stx17_Branchiostoma_lanceolatum
MSSFDDFEESLKRPVADDTPKQPIKILEPALKNFTKIAIPHILEKLNTHKINIEKRSGKWDKLNAEQI
DASRSN

>XP_001629417.1_Stx1_Nematostella_vectensis
MRDRLDFLRAEDNTRDDGPPEYEDSIAIPMGGEFMDDFFQQTANIRENIDKIAQDVERVKKAHSAVLS
SAVPDQEVKDNLEICMSRIQKTANTVRSRIKAMEQQIKEDEKQGGSLHNNYAEARIKKCQHATLSRKF
IEVMSEYNTTQTEYRELCKARICRQLEITGKSKTSEEVEDMLESGNPSIFTSDIVIQTQQAKQALGDI
EARHRDIITLEKNIQELHEMFQDMYMLVESQGEMIDRIEFNVEQAVDYVQSAKTDTKKALTYQSKARR
KKILIIICCLILLAIIIGAIVGALNG

>XP_009051819.1_Stx1_Lottia_gigantea
MFPSLIRIWSSKAQSDDDDEHEVAVNVDSGGFMDEFFEQVEEIREMIDRIAYNVDEVKKKHSAILSAP
NTDDKMKEELDDLMSEIKKSANKVRAKLKVIEQNIEQEEHSNKSSADLRIRKTQYSTISRKFVEVMTG
YNAAQLDYRERCKARIQRQMEITGKTTTNEELEDMLESGNPAIFTQGASTTFIGS

>XP_006821089.1_Stx1_Saccoglossus_kowalevskii
MKDRLPALKASQSDEDDMPDDVAVNMDDGRFMEEFFKEVEEIRIHIDQIQSNVEEMKKKHSKILSSPQ
PDEKVKEELEELMADIKKTANKVRGKLKVIESGIEQEDIKSSADIRIRKTQHSTLSRKFVEVMTEYNG
TQTEYRERCKGRIQRQLEITGKTTTNDELEDMLESGNPAIFTSGIIMDTQQAKQALGDIEARHNDIIK
LENSIRELHDMFMDMAMLVEQQGEMIDRIEYNVEQSVDYVETAKMDTKKAVKYQSKARRKKIIILICC
VILGVVIAATFGGVFG

>XP_002734118.2_Stx1Lb_Saccoglossus_kowalevskii
MQDFFSEVHNIRGDINHIKERVDDMKKKHSDILSSPQPKPEDQAEVDRIMKEVKKVSNNVRMKLKEIE
KSIEEDKLQHNLKSPADLRIRKNQHSSLHYSFLQVMTEYSNSQVEYREKCKGRIHRQLEITGQNTTDE
EIEEMLETGNAAIFTSSIIADTQQAKQALGDIEARYDELMKLEQSIKELHEMFLDMAMMVEQQGEMID
SIEHNVEEAAEYVAQAEVATKSAVKYQSKARRKKIIIIAIIAIAITVIVLLIVFL

>XP_006815743.1_Stx1La_Saccoglossus_kowalevskii
MSRVSLSCWWRNQCMGIQRCYFQIVCCLISHSVTILNGFSSTEGFKGDTTGLGLPESGVVAQLIKDDD
ESVATDDGGKVRIDIEQLDSGENFMQEFFAQVNNIRGDIDHIKKCVNDMKKKHSEMLSSSKPKPEDQK
EVDRTMKEVKAVSNKVKANLKEIEKSIEEDKLQHNLKSPADLRIRKNQHSSLHYSFLQVMTEYGNSQV
EYREKCKGRIHRQLEITGQSTTDEEIEGMLEKGNAAIFTSSIIADTQQTKQALGDIETRYDELMKLEQ
SIKELHEMFLDMAMMVEQQGEMIDNIEHNVEETAEYVDHAVATTKSAVENQSKARRKKWIIITIMTVA
IIVIVLFIVLL

>XP_006812573.1_Stx12_Saccoglossus_kowalevskii
MMEEEIDLEALRERETSLRKLESDILDVNQIFKDLATMVHEQGDMVDSIEANVDSAQIHVEEGTQQLQ
KARDYQKKARKKMIIFVIILIIVIAVIGLIIYFSVR

>XP_002733358.1_Stx5_Saccoglossus_kowalevskii
MTTRRRRTISDEARDEDNLYNLNYSNKPYVSETYVDNSDMTCRDRTHEFLSAVKSFQSRQGNGVAKFS
QNNKLLLQRSEFTQIAKKIGHDISNTFAKLEKLTILAKRKSLFDDKPIEIQELTYIIKQDINNLNKQI
AQLQQLVKLRAHKNGRHMQSHSSQVVVSLQSKLASMSNNFKEVLELRTRNLKEQKTRRDQFSQGPVAA
SMPPSATKGNTGSVLLQDEKTSYGGLGGDVSINMEDMDKQRYQQQLQLIDEQDSYIQSRASTMENIEA
TIVELGSIFQQLAHMVKEQEEQVQRIDQQIDDTHGNIEAAHGELLKYFQSVTSNRWLMIKIFVVLLVF
FIVFIVFMA

>XP_002735572.1_Stx6/10_Saccoglossus_kowalevskii
MSLEDPFFVVKDEVQKAVSNAKSLYQRWCELLEDPNAVSKEEYDWTTNELRNSLRSIEWDLEDLEETI
GIVETNPRKFKIDSSEIHERKQFVVHTKDMVKDMKEHMASPSTKTREDRKTRTTLLPNGPKKGQDKYT
RLDNEMDRSNQRFIDDTRQQQQLVMEHQDDQLERVGDSVTVLKSMGQTIGNELDEQAVMLDDFATEME
RTDSKLDGVMKKMAKVTRMSNDRRQWTAIVVLIVIMIIVIVLFFLL

>XP_001345316.3_Stx1a_Danio_rerio
MKDRTQELRHGKELEEQNEVTMNNKMVIEDGFMDEFFEQVEEIREFIDSLAEKVGEVKRNHSATLASP

NPDEKTKTEFEELMNDIKTLANKVRSRLQHIQQSIEHEEAFNGQSADVRIRKTQHSTLSRKFVEVMSE
YNAAQSEYRERCKGRIQRQLEITGRKTTKEELETILESDNPSIFTTGVFMDCSITKQAMNEIETRHNE
IIQLESCIRELQDMFVDLAVLVENQGELINNIETNVSSAQEYVEKAKEETKAAIKIQKTSRTKLILIG
GCVSVCVLILIIALIFGLT

>NP_571598.1_Stx1b_Danio_rerio
MKDRTQELRSAKDSDDDEEVVHVDRDHFMDEFFEQVEEIRGCIEKLSEDVEQVKKQHSAILAAPNPDE
KTKQELEDLTADIKKTANKVRSKLKAIEQSIEQEEGLNRSSADLRIRKTQHSTLSRKFVEVMTEYNTT
QSKYRDRCKDRIQRQLEITGRTTTNEELEDMLESGKLAIFTDDIKMDSQMTKQALNEIETRHTEIIKL
ENSIRELHDMFVDMAMLVESQGEMIDRIEYNVEHSVDYVERAVSDTKKAVKYQSQARKKKIMIIICCV
ILGVVLRSSIGGTLGF

>XP_003199177.1_Stx2b_Danio_rerio
MRDRLADLNVGRHNASEDGGVAVSMERNDFMQDFFQKVEEVRRVIEKISSLVDEVKKKYSVILSAPNP
DEKTKEELEQLTVEIKKHANYVQKSLKSMHQSLPSDEQVNQASVDARIQKTQYTNLSHKFVEVMTQYN
EAQVSFREKSKSRIQRQLEITGKITTNEELEEMLETGNPSIFTSDIISDSQITRQALNEIESRHQDIL
RLESSIKELHDMFVDMAMLVETQGEMIDNIEKNVHNAVEYVGQAKVETKKAVRYQTRARRKHIILALI
VLVVVAVVALIIGLSVGLSGKTSTNTAAASSNAANPV

>NP_001003561.1_Stx2a_Danio_rerio
MKDRLGELSAHYNDITIDVESDAFLEKFLPKVDEAQKLIERLSFLVEEVKLRHRTILTEINPQAYVRD
ELELFGNDIKQIADAIQVKLKGGNVMTEEETEDLLQNKSPAVFTSNISSGSSITGQALNEIESRHKDI
RCLEASIQELHNMFTDIAMLVNSQGEMANNIAKTVMKTGNYVDQGKENIKQATEYKKSWRIRLPPLPS
FKRKAKPVTNEGLLNES

>XP_021336668.1_Stx3a_Danio_rerio
MKDRLEQLKATCDHDDEDVEIAVDNAAFMDEFFSQIEDIRNSIDKIDENVAEVKKLYSVILSAPTSDQ
KTQDDLEALTNDIKKMANNARNKLKTIERNLETEEVERVSADMRIRKSQHAVLSRKFVDVMTKYNEAQ
VDFREKSKGRIQRQLEITGKATTDEELEEMLEGGNAAVFTAGIVDSGISKQALSEIEARHKDIVRLES
SIKELHDMFVDIAMLVESQKMIIVGVVVAVVLVIILIIILTQVL

>NP_001295756.1_Stx3b_Danio_rerio
MKDRLEQLKSKSDQTADDVEIPMENKEFMDEFFAQIEEIRTSIDKIDENVVEIKRLYSVILSAPTSEQ
KTQDELEAVTNEIKKLANNARNKLKSIEQNLAANTEERVSADMRIKKSQHAILAKKFVEVMTKYNEAQ
VEFREKSKGRIQRQLEITGKATTDEELEEMLDGGNAAVFTAGIMDSGISKQALSEIEARHKDIMRLES
SIKELHDMFVDIAVLVENQGSMIDRIESNMDQSVGFVERAVADTKKAAKFQQEARRKKMMIMLCCTIL
AVIGGSLVYSWLT

>NP_956515.1_Stx4_Danio_rerio
MRDRTKELGNTADASDEDEETVALMIKPGSGTSANEDKENEAFFKKVQEIREGLETLKRKVSELENKQ
KTVLGVALPEDSMKKELQSLREDIKGMASQIQKKLKSLEPKKLEVEEKYIPVNVRMRRTQHGVLSREF
LELMGRCNTIQAQYRDRNVERIKRQLKITGNSVSDDELETMLESGQTDVFTQNILNDAKATRQALNEI
ESRHDEIIKLERSIKELHDMFQYLAMEVEAQGEMVDRIESNIKMSHDYVEKAVAETEAAVKTSKKVQK
KKIYIAVCLAVLLLIIAICLAISFS

>NP_001002333.1_Stx5a_Danio_rerio
MTCRDRTLEFQSACKSLQGRQLQNGTHSKPANNALKQRSDFTLMAKRIGKDLSNTFAKLEKLTILAKR
KSLFDDKAVEIEELTYIIKQDINSLNKQIAQLQDLVRSRSGQNGRHIQTHSNTIVVSLQSKLASMSND
FKSVLEVRTENLKQQRSRREHFSQAPVSASPLLANNFNSSVLMQDESRSLGAEVAIDMDSRANPLQLQ
LIDEQDSYIQSRADTMQNIESTIVELGSIFQQLAHMVKEQEETIQRIDANVDDTELNVEMAHGEILKY
FQSVSSNRWLMIKIFLVLIVFFVIFVVFLA

>NP_955924.2_Stx5b_Danio_rerio
MNTRRRHGPKSSQDGVYTGPSQTQSQLVQQLETPLAVPAPIAPVPDTFSMSCRDRTGEFQSVCKSLQG

RQNGAQPVRAVNNAIQKRSDFTLLAKRIGRDLSNTFAKLEKLTILAKRKSLFDDKATEIDELTYIVKQ
DINSLIKQIAGLQELVRSRSAQNGRHLQTHSNTIVVSLQSKLASMSSDFKSVLEVRTENLKQQRSRQE
QFSQTPASASAFHTNSFNNSVLMQDDSKKTDISIDMDLNSSQQMQLVNERDSYIQNRADTMQNIESTI
VELGSIFQQLAHMVKEQEETVHRIDANVEDTQLNVDLAHTEILKYFQSVSNNRWLLIKMFLVLVIFFI
VFVLFMT

>NP_001017879.1_Stx6_Danio_rerio
MSMEDPFFVVKGEVQKAVNTAQGLHQRWIELLQDAGGASKEEVDWTTNELRNSLRSIEWDLEDLDETI
SIVEANPKKFNLDAMELAKRKAFITSTRQTVREMKDHMTSPMAITVPEKKNRQTLMGEGGSRGPIWQP
SGEKYTRLDNELQTANSQFIEEQQTQQQLIAEKQDEHLELVSGTIGVLKNMSQRIGQELDEQAVMLDD
FSHEMDSTQSRLDNVMKKLAKVSHMTSDKRQWCAIGVLLAILFVVILLFIIL

>XP_003200808.1_Stx7_Danio_rerio
MYGSREVDANGLAQTISSNIQRITLLTNEIQQLMRHLGTAQDTSDLRQTLQEKQQSVNQLAKVTDKCM
KDFSSLPATTEQRQRKIQRERLITEFSNVLAVFQKAQREVAKKEKEFVARVRASSRVSGGDIDDVFGR
AAPAFQSEFSAQAQSYEENITEEDLRLIQERESSIRQLESDITDINEIFRDLGMMVHEQGDMIDSIEA
NVSNAEISVQSATEQLQRAAGHQTSFRKKIFILVAVLAVAALIIGLIIYASVSK

>NP_956669.1_Stx8_Danio_rerio
MSKDLWLENYDAACRLAQEIAENIHERNRQQRTGGNPAKINMTLRASLQKLKQNIAQLRETLNRAAVQ
RHIMQAEADRRQSLVDDLASRETRLNASFKGDITEAEPSRSTLMAGGNGSGSAVNPWLINESEETKGL
SFGEIKNQQQQIIEAQDAGLDALASVLSRQKQMGQEIGNELDEQNEIIDDLAQLVDKTDGRIKNETKR
VKLLDSKSASCGMMVVIVLLLIAIIVVACWQ

>NP_001313376.1_Stx10_Danio_rerio
MSMEDPFFVVKGEVQKALSKAQGLYERWEELLQEETPVSRDELDWSTNELRNCLRAIDWDLEDLHETI
SIVEANPGKFRLGEHELQERRDFVERTRKSVQLMKEQLSSPSAVAQAEKKNKQALLGATAKDRYAGLE
PHLVSANSRYIQEQQEQQQLIMQDQDEHLELVTGSIRVLKDMSSRIGDELDEQAVMLGEFNEEMDQTG
SRMDSVLKKMEKVSHMTSSRRQWCAIGVLVIILIVVLILFFAI

>XP_697581.4_Stx12b_Danio_rerio
MSYGMMDSSHSQSQPRDFNNLTQTCSSNIQKITQNTGQIKSMLFQLGTRPDTPELQDRLQQVQHYTNQ
LAKETNRHLKDLGTLPQPQSPSEQRQQRIQKDRLMNDFSAALNNFQVVQRRAAERERESVARARAGSR
FQVDELNQDEQLVTFEKNEGWRMQTEEEPVTEEDLELIKERETNIRQLESDIMDVNQIFKDLAVMIHD
QGDMIDSIEANVESAEVHVERGAEQLQHAAYYQRKSRKRMCILALVLSLVATIFAIIIWQAIR

>NP_001002051.1_Stx12a_Danio_rerio
MSYGRTDQRASPKDFSSLIQTCSSNIQKITLNTAQIKGLVNQLGTKLDTSGLRERLQYMQHHTNQLAK
ETNKHLKDLGSISLPVSLSEQRQQKIQKDRLMNDFSAALNNFQAVQRQAAEKEKESVARARAGSRLSA
DDGGHDEQLVSFDNNDDWGKTTTQTEDVAITEEDLELIKERETAIRQLESDILDVNQIFKDLAVMIHD
QGEMIDSIEANVESAEVHVERGAEQLQRAAQYQQKSRKKICFLAVGLSIVVLIIGIVIWKLS

>XP_005172176.1_Stx16_Danio_rerio
MATRRLTDAFLLMRNNAIQNRQILAEQLADDRMALVSGISLDPEAAIGVTKRLPPKWVEGVDEIQYEI
TRIRQKMKELASLHDKHMNRPTLDDSSEEEHAIEITTQEITQMFHRCQRAVTGLQTQSYHCTEQENRL
LTNVVSSLAQSLQELSLNFRHTQSGYLKRMKNREERSKHFFDSGPLVEEDEDIALYDRGFTDDQLVLV
QQNTVMVEERERE IRQIVQSISDLNEIFRDLAGMVVEQGTVLDRIDFNVEQSCVKTEEGLQQLQKAEQ
YQKKNRKMLVILILFVIVVVLILILFGTKFS

>NP_001007450.1_Stx17_Danio_rerio
MADEAGKLPLRRLEPPIQKFIKVAIPTDLERLHQHQHNIEKFQRNRQWDKLHHEHINSSRTVQQLRSN
LREMEKLCGRVRSVDAEALEKLVQPIRDRASAAIQEFLQIHSDAVNRQNFNEAIATVAETSHSEDDTG
VSGSPVTQTQLLLPEIPSEQNAAESWDSLAEDLLQLNGLVNEFSTIVYAQQEKIDSIEANVSIAAANV
EEGTQSLGKAARAKLAVLPVAGAVVGGVLGGPLGLLAGFKVAGVAAAFGGGLLGFAGGNLIQRKRKER

IDLQLQELHSNNNSKNDTQ

>NP_001019596.1_Stx18_Danio_rerio
MAADITLLFKASVKTVKTRNKALGLMETTGREEPGAQKRPRPRQRDSFSCKAREVISNISKLKDFLLQ
HRKDYVNAGSVMSSEVSRMTDSERDQIDQDAQIFMRTCAEAISQLRSEMDKKQVSMQVKDHRAAVLDL
IDSYLKGVCKLYSEQRAVRVKRVVDKKRLSRLEPEQINTEKRQSDGEENDKPVSDGNVDLWEENRVED
DLSPEEIQMFEQENQRLVGEMNSLLDEVRQIEGKVVEISRLQEIFSEKVLQQETEIDSIHQLVVGATE
NVKEGNEDIREAIKNNAGFRVWILFFLVMCSFSLLFLDWYDS

>XP_021334545.1_Stx19_Danio_rerio
MKDRMEELRQQMKASEKLVDSKPFNKEEDEDMDPSSLIGLQAVIFEAEPVLEIFLKDAQGIRDSIEEL
NSEVSKFNEQQKNFVATMRRLSIMKKESNMTRDIKLLAESLHKRLDALSKQAKQTEAELGPNATTSRI
QKIQHAALFLQFHQVMRQHNDAILSKQEKCKQFIIRQLEVSGREVSEEEVDNMIEQGKWEIFNENIIV
DAKITRTQLSEIEQRHKELLNLESNMKDLRDLFLDVFMLVEEQGHQIQNIQANVEKTQDYVSVTKEKF
KRAARYKKNNPLRRLCCCCCPWFR

>XP_015223056.1_Stx1a_Lepisosteus_oculatus
MDSNITKQAMNEIETRHNEIIKLENSIRELHDMFMDMAMLVESQGEMIDRIEYNVEHSVDYVERAVSD
TKKAVKYQSKARRKKIMIIICCVVLGIVIASSLAGTLG

>XP_015201626.1_Stx1b_Lepisosteus_oculatus
MKDRTQELRSAKDSDDDEEVVQVDRDHFMDEFFEQVEEIRGCIEKLSEDVEQVKKQHSAILAAPNPDE
KTKQELEDLTADIKKTANKVRSKLKAIEQSIEQEEGLNRSSADLRIRKTQHSTLSRKFVEVMTEYNTT
QSKYRDRCKDRIQRQLEITGRTTTNEELEDMLESGKLAIFTDDIKMDSQMTKQALNEIETRHNEIIKL
ENSIRELHDMFVDMAMLVESQGEMIDRIEYNVEHSVDYVERAVSDTKKAVKYQSKARRKKIMIIICCV
VLGVVLASSIGGTLGF

>XP_015221738.1_Stx2_Lepisosteus_oculatus
MRDRLADLTASSKYEDAECSVAIERDAHMDSFFKKVEEVRSTIDKISNEVEEVKKKHSIILSAPNPED
KTKEELEQLTIEIKKNANAVRARLKSMEQNLDQDDNANRSSVNYRIQKTQHTILSRKFVEVMTQYNDT
QVSFRERSKGRIQRQLEITGKVTTSDELEDMLESGNPAIFTSDIISDSQITRQALNEIESRHQDIMKL
ECSIRELHEIFTDMAMIVETQGEMINNIEKNVTNAAEYVGRAKEETKKAVKYQSKARRKYLIIAIAVL
VLLGIIALIVGLSLGLK

>XP_006630262.1_Stx4_Lepisosteus_oculatus
MRDRTRELGDKAESSDEEEEGRPLMIKPGTGKTSQKAAKDENDEFFQKVREIHEGLAALTMKVNSLEV
KQRTILGVALPEESMKRDLQALRDEIKMSASHIQKKLKSIEPKKEEEDGKYVPVNVRMQRTQHGILSR
EFVELMGRCNAVQSQYRDRNVERIQRQLKITGTNVTDEELDQMLESGQTDVFTQNILRDTHATKQALN
EIETRHEEILKLERSIRDLHEMFQYLAMEVEAQGEMVNRIESNILQSSNYVEKATADTEKAKEYHVKS
QKKKLCIAVCVIVLILVIIAILAGTFAS

>XP_006642820.1_Stx5_Lepisosteus_oculatus
MYTRRRHGSRSTEQGVYLGPSQTQAQDCPPPAGPPPLPVATDNAMSGRDRTQEFLSACKSLQGRQNGV
QVHKPALSAVKQRSDFTIMAKRIGKDLSNTFAKLEKLTILAKRKSLFDDKAVEIEELTYIIKQDINSL
NKQIAQLQDLVRSRGGQSGRHIQTHSNTIVVSLQSKLATMSNDFKSVLEVRTENLKQQRSRREHFSQA
PVSSSPLHANNFGSSVLLRDDSPRSADVSIDMDARASQQLQLLDEQDSYIQSRADTMQNIESTIVELG
SIFQQLAHMVKEQEETIQRIDANVEDTQLNVEAAHGEILKYFQSVTSNRWLMVKIFLILIIFFVVFVV
FLA

>XP_015211993.1_Stx6_Lepisosteus_oculatus
MSMEDPFFVVKGEVQKAVNTAQGLYQRWTELLQEPGSATKEEVDWTTNELRNSLRSIEWDLEDLDETI
SIVESNPRKFNLDTMELTKRKAFITGTRQTVKEMKDHMSSPMAQAMSDRKNRQALLGESVSQGPGWHS
GTDKYTRLDRELQSANSQFIEDQQAQQQLIVEQQDDQLELVSGSVGVLKNMSQRIGQELDEQAVMLDD
FSHEMDNTQSRLDNVMKKLAKVSHMTSDRRQWCAIGILLAILLVVLILFFAL

```
>XP_006626240.1_Stx7_Lepisosteus_oculatus
MSYQSGIPRDANQLAQAISSNIQKITHQTSEIQRIINQLGTPQDTTELRQKLQQKQQNVSHLAKETDR
YMKEFGSLPVTSEQRQRKIQKDRLLNDFSNALAAFQKIQRQAAQKEKEFVARVRANSRISGGLPDDGF
KGESSSPFESGGQPQAQVQDDVITEEDLMLIKERETSIRQLESDIMDINEIFKDLGIMIHEQGETIDS
IEANVENADVHVQTATQQLARAAEYQRKSRKKICVIIVVLTIVAVIIGVIIWVSVKK

>XP_015212214.1_Stx8_Lepisosteus_oculatus
MSQDPWLQVYDATCRLAQEIAENIHERNRQHRTGGNPAKLNMTIRASLQRLKQQIAHLRERLLTSTST
RRIMQSEGDRRQNLLDDLLTREEQLDASFNKDGTEPDSSRSSLLTGGMDPAGLGNPWLINEPEETRGL
GFGEIKQQQQKIIEVQDAGLDALAAVISRQKQMGQEIGNELDEQNEIIDDLTHLVDHTDGRIRKETKR
VKIVETKSTSCGMLVVIVLLLIAIVVVAVWPTK

>XP_006631337.1_Stx12_Lepisosteus_oculatus
MSHGRDAYRSQPRDFNTLIQICSSNIQKITQNTGQIKAMVNQLGTRQDTSELQDKLQQLQHYTNQLAK
ETNRHLKDLGSLPLPLSPSEQRQQKIQKERLMNDFSAALNNFQAVQRRAAEKEKESVARARAGSRLSA
EDSNRDEQLVSFDTNDEWGQTQDQTEEAAITEEDLELIKERETNIRQLESDIMDVNQIFKDLAVMIHD
QGEMIDSIEANVESAEVLVERGTDQLHQASHYQKKSRKKMCILALILSLVVVILGIIIWQASK

>XP_015220591.1_Stx16_Lepisosteus_oculatus
MATRRLTDAFLLMRNNAIQNRQILAEQLADDRMALVSGISLDPEAAIGVTKRLPPKWVDGVDEIQYDI
TRIRQKMKELASLHDKHMNRPTLDDSCEEEHAIEITTQEITQMFHRCQRAVAALQRRRGHCTEQEERL
LKNVVSSLAQSLQDLSTGFRHTQSSYLKRMKNREERSKHFFDTSGPLVEDDDDNTLYDRGFTDDQLAL
VEQNTILVEEREREIRQIIQSISDLNEIFRDLAGMVVEQGTVLDRIDFNVEQACVKTDEGLKQLQKAE
QYQKKNRKMLVILILFVLVVVLILVLIGVKFT

>XP_006635799.1_Stx17_Lepisosteus_oculatus
MADEAGSLPLRRLEVPIHKFIKVAIPTDLERLRKHQINIEKYQRCQQWDRLHQEHINASRTVQQLRAN
IREMEKLCARVRSEDTRALDRLVRPTREQASVAAGDFLRLHSETAAPTGVQPTGPESPPAPSLSRSLT
ALGSFSGGEESSIPLQTQTQLPLPEIPRDQNAAESWETLEEDLLELNGLVNEFAELVHSQQEKIDSIE
DHVNTAAANVQEGSRSLGQAARYKMAMLPVAGAVIGGVVGGPLGLLAGFKVAGVAAAVSGGLLGYAGG
NLLQKSRRAKVDTQLQQLSTSCPELCKQEDKKKQ

>XP_006629663.1_Stx18_Lepisosteus_oculatus
MAVDITLLFKASVKTVKTRNKAIGVGFDSTKDDILKKTRLKSDFSTKAKEVISNITKLKDFLLQHRKD
YVNAGSLISSDYTRMTDNERDQIDQDAQIFMRTCSDAINQLRTEADKKVMSAQVKEHRGAVLDLIEDY
LKGVCKLYSEQRAIRVKRAVDKKRLLRLEPERHSKLLSSATEKSAQPDPSDEKTLKDNTTDTNVAEAQ
ESGVGLWEDGKVEDELSPEEIQMFEQENQRLVSEMNSLVDEVRQIEGKVVEISRLQEIFAEKVLHQET
EIDNIHQLVVGTTENVKEGNEDIREAIKNNAGFRVWILFFLVMCSFSLLFLDWYDS

>XP_015217154.1_Stx19_Lepisosteus_oculatus
MKDRLEELRQRAKDAEMEKEKNTFADDVTDGASFSPQAVVFETEPGIENFLSEVQRIRDSINDLADEV
KRFSQQQKNLVATMRRFSVMKKESSVTRDIKLQAESLHRKLDALAKRVKSTEAEHGPNAAITRIQSTQ
YSTLFRHFQQVMRQYNDTLVSKQDKCKQFIVLQLEVAGKEVSEDEVDEMVEQGKWEVFNENVLNEVKI
TKAQLTEIEQRHKELMNLESNMKDLRDLFLEIYLQVEEQGEHIENIESNIQKTQDFVQKTNEKFKLAA
KYRKKNPLKKMCCCCCPCCK
```
